# Chronic infection perturbs the affinity hierarchy of antiviral B cells

**DOI:** 10.1073/pnas.2532422123

**Published:** 2026-03-31

**Authors:** Mirela Dimitrova, Tiago Abreu-Mota, Jonas Fixemer, Weldy V. Bonilla, Anna-Friederike Marx, Min Lu, Karen Tintignac, Anna Lena Kastner, Yusuf I. Ertuna, Marianna Florova, Matias Ciancaglini, Kerstin Narr, Karsten Stauffer, Julien Roux, Philippe Demougin, Ingrid Wagner, Doron Merkler, Daniel D. Pinschewer

**Affiliations:** ^a^Department of Biomedicine, Division of Experimental Virology, University of Basel, Basel 4009, Switzerland; ^b^Department of Hematology, Oncology and Cancer Immunology, Charité-Universitätsmedizin Berlin, Freie Universität Berlin and Humboldt-Universität zu Berlin, Berlin 13353, Germany; ^c^Max-Delbrück-Center for Molecular Medicine and Berlin Institute of Health, Berlin 10115, Germany; ^d^Department of Biomedicine, Bioinformatics Core Facility, University Hospital Basel, Basel 4031, Switzerland; ^e^Genomics Facility Basel, Pharmacenter, University of Basel, Basel 4056, Switzerland; ^f^Department of Pathology and Immunology, University of Geneva, Geneva 1211, Switzerland; ^g^Division of Clinical Pathology, Geneva University Hospital, Geneva 1206, Switzerland

**Keywords:** persistent viral infection, immune subversion, B cell, affinity maturation, lymphocytic choriomeningitis virus

## Abstract

A key function of the germinal center (GC) reaction consists in the preferential expansion and enrichment of high-affinity B cell clones. Whether and how persistent viral infection thwarts this purpose remains ill-defined. Here, we transferred monoclonal lymphocytic choriomeningitis virus–specific B cells into persistently infected hosts. High-affinity B cells expanded vigorously, forming GCs and abundant antibody-secreting cells. When failing to clear viremia or to drive viral mutational escape, the expanded B cell population contracted, ending in its quasi-complete disappearance from the spleen, a process we termed “attrition.” In stark contrast, low-affinity B cells expanded and persisted irrespective of high viral loads. B cell attrition was associated with phenotypic and transcriptional alterations including a prominent Blimp-1 transcriptional signature in high-affinity GC B cells. Blimp-1-deficient B cells were resistant to attrition, suggesting a B cell–intrinsic process. Moreover, exogenously supplied antibody feedback prevented attrition, suggesting that the latter may have resulted from excessive antigenic stimulation. Our findings suggest that in chronic viral infection, the incessant exposure to overwhelming amounts of antigen perturbs B cell affinity hierarchies by preferentially dysregulating high-affinity B cells.

## Significance

Chronic viral infections pose a major challenge to global health, yet how viral persistence undermines antibody-mediated immunity is insufficiently understood. Here, we report that under continuous exposure to abundant viral antigen, high-affinity antiviral B cells undergo vigorous expansion followed by near-complete clonal deletion in the spleen—a process we term “attrition.” In striking contrast, low-affinity B cells sustain long-term responses throughout chronic viral infection. We further show that attrition is a B cell–intrinsic process that can be prevented by exogenously supplied antibody through a negative feedback–like mechanism. These findings offer an explanation for the long-standing observation that neutralizing antibody responses to chronic viral infections are often delayed and quantitatively inadequate, and they highlight potential avenues for therapeutic intervention.

The World Health Organization estimates that more than 300 million people worldwide live with hepatitis B and/or hepatitis C virus (HBV, HCV) (1, 2), and approximately 38 million people are carriers of HIV (3). Despite potentially fatal long-term consequences, a functional cure for HBV or HIV infection remains currently out of reach (4, 5). Chronic viremic infections tend to subvert adaptive immune defense as exemplified by “T cell exhaustion,” which is intimately linked to persistently high antigen levels (6, 7). Evidence is accumulating that not only T cells but also humoral immune responses are suppressed in chronic viral infection. This is evident in delayed and inadequate antibody responses to HCV and HIV in humans and to lymphocytic choriomeningitis virus (LCMV) in mice (8–10). The B cell compartment in chronic HBV and HIV infection undergoes phenotypic alterations as reflected in the accumulation of atypical memory B cells (MBCs) and plasmablasts, suggesting biased end-differentiation (11, 12). Additionally, B cells in the peripheral blood of patients exhibit elevated levels of inhibitory receptors such as FcRL4 and PD-1 (11, 13, 14) and in the case of HBV infection show impaired differentiation into antibody-secreting cells (ASCs) as well as inadequate immunoglobulin secretion (13, 14). We and others have reported that type I interferon (IFN-l) responses at the onset of chronic infection can lead to so-called *decimation* of virus-specific B cells (15–17), reducing antiviral B cell expansion by ~10-fold (18). While contributing to delayed neutralizing antibody (nAb) responses, the aforementioned effect size seems insufficient to fully account for the inadequacy of antibody responses to chronic viral infection, necessitating further investigations into the limitations of B cell responses in this context. B cell–based protection has significant potential as a means to contain persistent viral diseases. Timely nAb responses upon primary as well as secondary HCV infection herald spontaneous immune-mediated viral clearance (19, 20). Similarly, high frequencies of HIV-specific MBCs are a frequent characteristic of HIV posttreatment controllers that suppress viral loads for several years after cessation of antiretroviral therapy (21). Moreover, potent anti-HIV antibody responses are often associated with HIV elite control (22–24) and spontaneous clearance of HBV carriage goes along with seroconversion to protective anti-HBs antibodies (5). Prominent glycan shields impede, however, antibody access to neutralizing epitopes on the envelope glycoproteins of HIV, HCV, and LCMV, rendering these viruses extremely challenging targets for nAb induction (25–27). Even when presented to the immune system in the context of vaccination rather than in chronic infection, nAb responses are weak or not elicited at all (10, 27). Accordingly, only a minority of HIV-infected individuals succeed after years of viremic infection in mounting broadly neutralizing antibodies (bnAbs) that cover a majority of viral genomic variants (28, 29). With rare exceptions, however, such bnAbs fail to neutralize the donating patients’ autologous virus at the time of antibody cloning (23). Known limitations curtailing bnAb responses comprise that in HIV-negative individuals only about one naïve precursor of bnAb-producing B cells is found per million B cells (30). Moreover, these germline antibodies bind to their target with only low to intermediate affinity and cannot neutralize the virus (31). Therefore and unlike for acute viral infections such as SARS-CoV-2, influenza A virus, or vesicular stomatitis virus (32–34), the evolution of HIV-bnAbs necessitates substantial affinity maturation (31). The continuous maturation of serum antibody affinity over time is the end result of somatic B cell receptor (BCR) hypermutation in the germinal center (GC) dark zone (DZ), followed by competition of B cells for antigen on follicular dendritic cells and the selection of high-affinity clones by cognate follicular T helper cells in the light zone (LZ) (35). Interclonal competition of B cells is not, however, limited to the GC. Even very-low-affinity clones can be recruited to GCs (36–38), but affinity-based pre-GC competition for T help can restrict GC access (37, 39), a limitation that is relaxed under conditions of abundant cognate T help (40). These several layers of Darwinian selection predict a preferential expansion and enrichment of high-affinity clones in the GC over time, alongside a quasi-complete elimination of lower-affinity competitors. Single-cell GC repertoire analyses from vaccinated animals have, however, revealed that the average BCR affinity increases over time while the GC reaction as a whole maintains a broad range of affinities throughout the response, notably including low-affinity BCRs (41–44). Chronic viral infection is predicted to set exceptional conditions for B cell selection, since antigen is available in excess and the GC response is tasked to convert B cell clones of very low abundance and low starting affinity into a high-affinity bnAb response (29, 31). It remains unknown but would be important to understand how these exceptional parameters impact affinity hierarchies in antiviral B cell responses.

Here, we report that in chronic viral infection, transient expansion of high-affinity B cells is followed by their quasi-complete disappearance, a process we termed “attrition,” whereas low-affinity B cells mount sustained responses. These findings suggest that persistently high levels of viral antigen in chronic infection can perturb the affinity hierarchy of responding B cell populations.

## Results

### Transient Expansion of Antiviral B Cells when Transferred at Very Low Numbers into Chronically Infected Hosts.

**Fig. 1.**
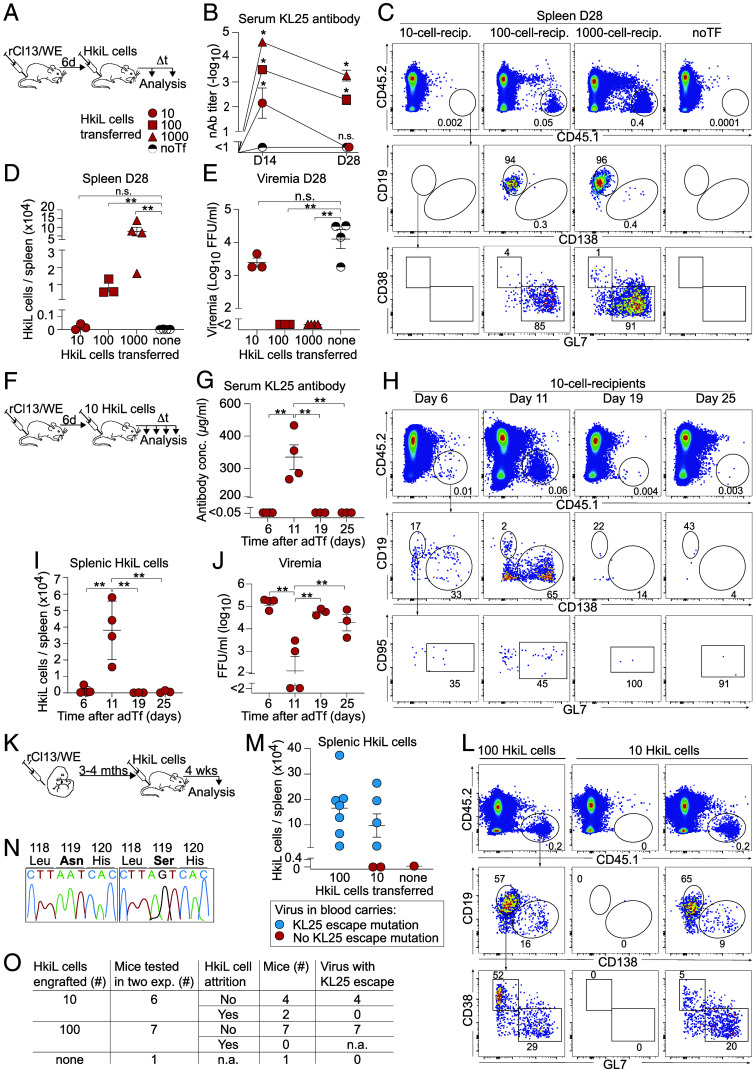
Transient expansion of antiviral B cells when transferred at very low numbers into chronically infected hosts. (*A–E*) We infected mice with rCl13/WE on d-6. On d0, we engrafted either 10, 100, or 1,000 HkiL cells or none (noTF), collected serum over time, and analyzed HkiL progeny in the spleen on d28 (*A*). nAb titers over time (*B*) and representative flow cytometry plots (FACS) plots from the spleen on d28 (*C*; 3 mice in 10- and 100-cell groups, 4 mice in the remainder groups) pregated on live lymphocytes (*SI Appendix,* Fig. S1*A*) are shown. HkiL progeny (CD45.1^+^, *Top*) were analyzed for expression of CD19 and CD138 (*Center*), and their CD19^+^CD138^−^ subset was analyzed for GL7 and CD38 expression (*Bottom*). Total CD45.1^+^ KL25 progeny counts (*D*) and viremia (*E*) were determined on d28. (*F*–*I*) We infected mice with rCl13/WE on d-6. On d0, we engrafted 10 HkiL cells. Serum was collected, and groups of mice were killed on the indicated days (n = 4 on d6 and d11; n = 3 on d19 and d25). KL25 antibody concentration was determined by enzym-linked immunosorbent assay (*G*) and CD45.1^+^ HkiL progeny (*H*) were analyzed as in (*C*) and enumerated (*I*). Viremia (*J*) was determined at the indicated time points. (*K*–*O*) In two separate experiments, we infected newborn mice with rCl13/WE. When they were 14 to 16 wk old we engrafted them with either 10 or 100 HkiL cells and analyzed them 3 to 4 wk later (*K*; 21 d and 31 d in the two respective experiments). Representative FACS plots (*L*; n = 7 for 100-cell group, n = 6 for 10-cell group) and total cell count (*M*) of splenic HkiL cell progeny. In (*M*), mice that harbored viruses with KL25 escape mutation are shown in blue, mice with viruses devoid of such mutations are displayed in red. Exemplary fluorograms of Sanger sequencing reactions from viruses in mouse blood (*N*). The sequence around Aa119 of the viral GP (*N*) and a summary table of experimental outcome in individual mice (*O*) are shown (*SI Appendix*, Table S1). Numbers in FACS plots indicate the percentage of gated cells (mean of the group). Symbols in (*D*, *E*, *G*, *I*, *J*, and *M*) show individual mice with the mean ± SEM indicated, in (*B*) they show the mean ± SEM (n = 3 to 4 per group). Data in (*A*–*J*) show one out of two similar experiments. Panels (*K*–*O*) report combined results from two experiments. Two-way ANOVA with Tukey’s post-test was performed in (*B*) reporting for each time point significant differences in comparison to the noTf group. One-way ANOVA with Dunnett’s post-test was performed in (*D* and *E*), compared to the noTF group. In (*G*, *I*, and *J*), we performed One-way ANOVA with Tukey’s post-test, comparing the means of all groups and showing only significant differences. *P < 0.05; **P < 0.01; n.s.: not statistically significant.

Considering the very low precursor frequency of bnAb-producing B cells in humans (30) and the challenges related to the efficient recruitment of such low-frequency B cell populations into GC responses to vaccination (45), we set out to study how very low numbers of nAb-producing B cells respond to chronic viral infection in mice. HkiL donor B cells are engineered to express the KL25 antibody, which binds the LCMV-WE strain envelope glycoprotein (GP) at 5 nM affinity and exhibits potent virus-neutralizing activity (46). Consistent with earlier reports (45, 47, 48), we found that the splenic take of adoptively transferred HkiL cells was in the range of ≤5% (*SI Appendix,* Fig. S1 *A*–*E*) (49). Taking this uptake rate as a basis of calculation, we engrafted either 10, 100, or 1,000 HkiL cells in the spleen of mice that underwent chronic infection with an engineered Clone 13-based LCMV strain expressing the WE strain GP (46) (rCl13/WE; Fig. 1*A*) (50). While this approach enabled a qualitative assessment of how precursor frequency influences the response of adoptively transferred HkiL cells to infection, the splenic uptake rate may depend on the absolute number of B cells transferred, such that the calculated number of engrafted cells should be considered an approximation. Adoptive B cell transfer (Tf) was conducted 6 d after virus administration to avoid IFN-I-induced *decimation* in the first few days after LCMV infection (15–17) and to mimic the continuous recruitment of new clones into ongoing B cell responses (51–53). 4 wk after engraftment of 100 or 1,000 HkiL cells, their expanded progeny populations (CD45.1^+^) were readily detected in the respective recipients (100-cell recipients, 1,000-cell-recipients) and displayed mostly a GL7^+^CD38^–^CD138^–^ GC phenotype (Fig. 1 *C* and *D* and *SI Appendix,* Fig. S1*A*). In contrast, recipients of 10 cells (10-cell-recipients) did not harbor HkiL progeny at numbers clearly exceeding technical backgrounds of mice without adoptively transferred HkiL cells (“noTf”). In keeping therewith, 100-cell-recipients and 1,000-cell-recipients but not 10-cell-recipients suppressed viremia to below detection limits at week 4 (Fig. 1*E*). The serum of 10-cell-recipients did not contain any detectable KL25 antibody at week 4 either, but the same was clearly detected at week 2, suggesting that HkiL cells mounted a transient antiviral response (Fig. 1*B*). Accordingly, a time course analysis in the spleen of 10-cell-recipients showed that HkiL cells expanded until day 11 after engraftment, forming a substantial population of predominantly ASC-differentiated (CD138^+^B220^low/int^) progeny, but contracted to background levels by day 19 and thereafter (Fig. 1 *H* and *I*). This initial expansion and subsequent collapse of the HkiL cell population was reflected in a transient KL25 antibody response in serum and a concomitant suppression of viremia on day 11, which rebounded by day 19 (Fig. 1 *G* and *J*). To extend and generalize our observations, we performed HkiL Tf experiments in neonatally infected carriers of rCl13/WE, which exhibit life-long unchecked viremia at levels that are equivalent to or higher than those observed when chronic infection is established in adult life (54). Neonatally infected carriers develop, however, antiviral CD8 T cell tolerance (55) and are virtually devoid of the antiviral inflammatory reaction (56), which in adult infected animals can profoundly impact antiviral B cell responses (15–17). When engrafting 100 HkiL cells into rCl13 carriers, all animals exhibited a clearly detectable population of HkiL cell progeny 4 wk later, most of which were GC B cells (Fig. 1 *K*–*M*). Interestingly, analogous populations were also found in four out of six carriers engrafted with 10 HkiL cells in two independent experiments, whereas one animal in each experiment was devoid of detectable HkiL cell populations. We found that in all 10-cell- and 100-cell-recipients with detectable HkiL cell populations, the virus persisted but had acquired typical KL25 escape mutations at amino acid 119 of the GP (46) (Fig. 1*M*, blue symbols; Fig. 1 *N* and *O* and *SI Appendix*, Table S1). No such mutations were found in animals that were devoid of detectable HkiL cell progeny (Fig. 1*M*, red symbols; Fig. 1 *N* and *O* and *SI Appendix*, Table S1). Altogether these observations suggested a dichotomous development of adoptively transferred HkiL cells in chronic LCMV infection. When transferred in higher numbers, these antiviral B cells eliminated the virus in adult rCl13/WE infection or drove viral mutational escape in neonatally infected carriers. When engrafted at critically low cell numbers, however, the transferred HkiL cell population underwent initial expansion but contracted again, ending in its quasi-complete disappearance from the spleen, a process henceforth referred to as “attrition.”

### Chronic Infection with High-Affinity but Not Low-Affinity Virus Antigen Causes B Cell Attrition.

**Fig. 2.**
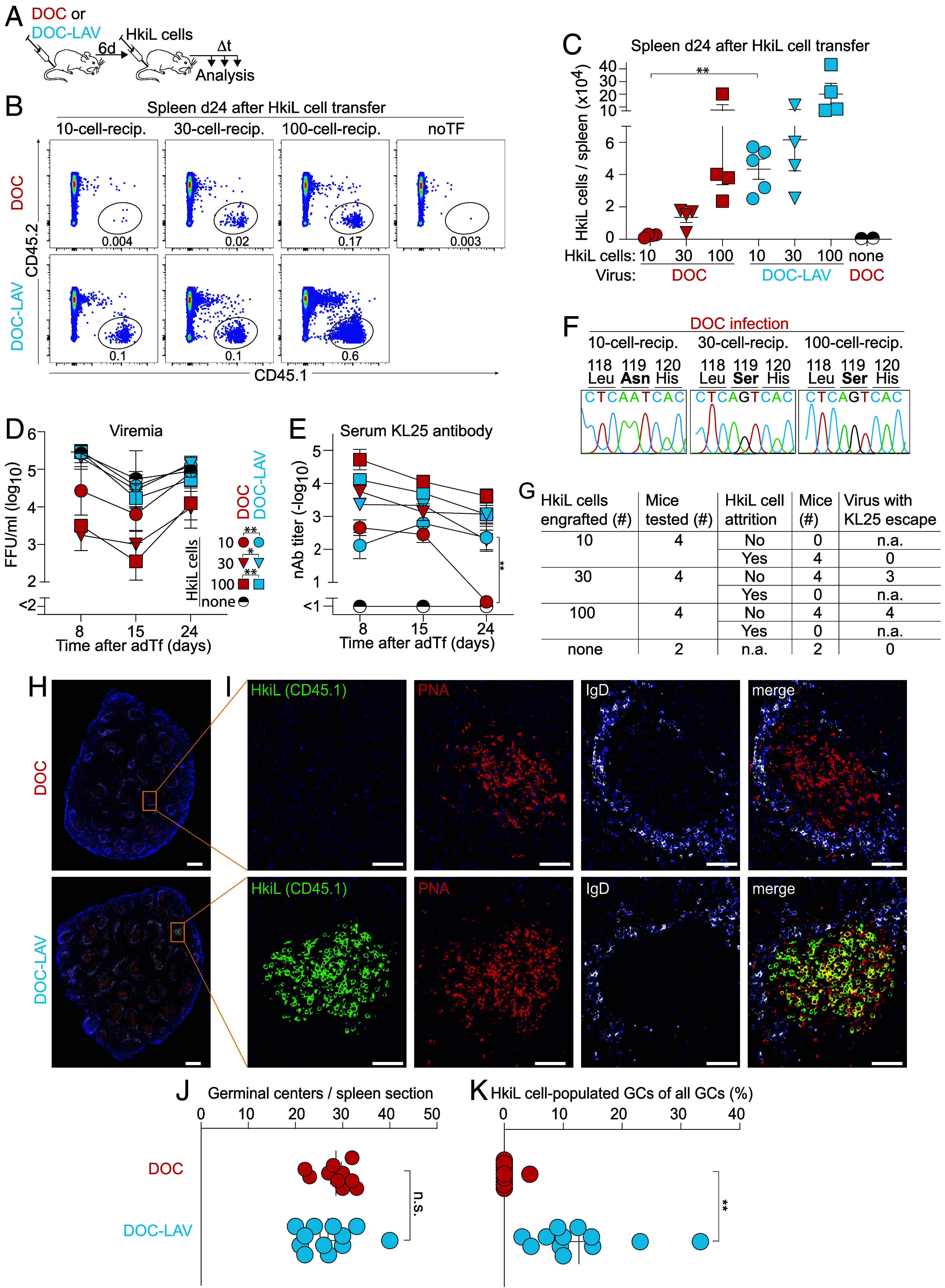
Chronic infection with high-affinity but not low-affinity viral antigen causes B cell attrition. We infected mice with DOC or DOC-LAV on d-6. On d0, we engrafted either 10, 30, or 100 HkiL cells or none (noTF), collected blood and serum samples over time, and analyzed HkiL cell progeny in the spleen on d24 (*A*–*C*). Representative FACS plots [*B*; n = 4 mice per group except DOC-LAV-infected 10-cell-recipients (n = 5) and noTf (n = 2)] pregated on live lymphocytes (*SI Appendix,* Fig. S1*A*) show HkiL progeny (CD45.1^+^). The cells’ expression of B220 and CD138, and the GL7/CD38 profile of the B220^+^CD138^−^ subset are reported in *SI Appendix,* Fig. S2*C*. HkiL progeny were enumerated (*C*) and viremia (*D*) as well as nAb titers (*E*) were determined over time. Exemplary fluorograms of Sanger sequencing reactions of viruses persisting in DOC-infected 10-cell-, 30-cell-, and 100-cell-recipients on d24 (*F*). The sequence around Aa119 of the viral GP and a summary table of experimental outcome in individual mice (*G*) are shown (*SI Appendix*, Table S1). Spleen sections were stained for CD45.1 (HkiL cells; green), PNA (GC; red), IgD (white), and DAPI (*H* and *I*). The total number of GCs per section (*J*) and the percentage of GCs containing three or more CD45.1^+^ cells (*K*) were determined. Numbers in FACS plots indicate the percentage of gated cells (group mean; *B*). Symbols in (*C*) show individual mice. Symbols in (*D* and *E*) represent the mean ± SEM of n = 4 to 5 mice per group (except noTf group: n = 2). In (*H* and *I*), representative images from 10 spleen sections of 4 to 5 mice per group are shown, symbols in (*J* and *K*) represent individual spleen sections (up to three sections per mouse taken at ~100 µm distance from each other) with the mean ± SEM indicated. Magnification bars: 500 µm (*H*) and 50 µm (*I*). One-way ANOVA with Tukey’s post-test of log-converted values was performed in (*C*), and significant differences between groups receiving the same number of HkiL cells are reported. Repeated measures ANOVA was performed for a pairwise comparison of DOC- and DOC-LAV-infected groups receiving the same respective number of HkiL cells (*D*). Two-way ANOVA with Tukey’s post-test was performed in (*E*) and the only significant difference between groups receiving the same respective number of cells is reported for the respective time point. Unpaired Student *t* tests were performed in (*J* and *K*). Only statistically significant differences are shown in (*C*–*E*). Panels (*B*–*F*) show one representative out of two similar experiments. *P <0.05; **P < 0.01; n.s.: not statistically significant.

The observation that HkiL cells persisted in neonatally infected carrier mice whenever the virus underwent mutational escape was intriguing, prompting us to study the response to N119S-mutant virus in the adult infection setting. These attempts were, however, encumbered by the inability of rCl13/WE to persist when more than 10 HkiL cells were engrafted. Hence, we established a reverse genetic system for the LCMV strain Docile (DOC) (57), a variant of the WE strain which establishes more robust and long-lasting viremia than rCl13/WE (*SI Appendix,* Fig. S2 *A* and *B*). We generated a pair of viruses, DOC [wild-type (WT) virus] and a KL25 low-affinity variant (DOC-LAV). DOC-LAV differed from DOC by only one single point mutation in the viral GP (N119S), an escape mutation without associated fitness cost that was frequently detected in the experiments to Fig. 1 *K*–*O* (*SI Appendix*, Table S1) and is known to reduce KL25 binding by ≥1,000-fold (46). Mice were infected with either DOC or DOC-LAV and were engrafted 6 d later with either 10, 30, or 100 HkiL cells (Fig. 2*A*). When analyzing spleens of DOC-infected mice 24 d later, HkiL cell progeny were readily detected in 30- and 100-cell-recipients but had seemingly undergone attrition in 10-cell-recipients (Fig. 2 *B* and *C* and *SI Appendix,* Fig. S2*C*). In stark contrast, a sizable population of HkiL cells persisted in DOC-LAV-infected 10-cell-recipients and consisted mostly of GC B cells (Fig. 2 *B* and *C* and *SI Appendix,* Fig. S2*C*). KL25 antibody production was readily detected in all groups of mice on day 8 and on day 15 after HkiL cell engraftment, but in DOC-infected 10-cell-recipients dropped to below detection limits on day 24 (Fig. 2*E*). This decline in serum antibody titers of DOC-infected 10-cell-recipients significantly exceeded the one in 30-cell- and 100-cell-recipients (*SI Appendix,* Fig. S2*D*), suggesting a disproportionate contraction of the HkiL cell response that was consistent with attrition. Analogously to the flow cytometric findings in the spleen, HkiL cells persisted in inguinal lymph nodes of DOC-LAV-infected but not DOC-infected 10-cell-recipients (*SI Appendix,* Fig. S2 *H* and *I*). Irrespectively of the infecting virus, however, HkiL cell progeny were not consistently detectable in the bone marrow (*SI Appendix,* Fig. S2 *E*–*G*), which was likely due to the well-documented destruction of the plasma cell niche-forming stroma during chronic LCMV infection (58–60). As hoped for and unlike in adult rCl13/WE infection, viremia persisted in all groups of mice throughout the observation period (Fig. 2*D*, compare Fig. 1*E*). Still, HkiL cells afforded partial suppression of DOC viremia, whereas replication of the KL25 escape variant DOC-LAV was unaffected, as expected (Fig. 2*D*) (46). Sequence analysis of the persisting DOC viruses on day 24 after HkiL cell Tf evidenced an unmodified DOC GP sequence in 10-cell-recipients but mutational escape in 30-cell- and 100-cell-recipients, correlating with the attrition and persistence of HkiL cells, respectively (Fig. 2 *F* and *G* and *SI Appendix*, Table S1). These analyses were complemented by immunofluorescence stains of spleen sections from DOC and DOC-LAV-infected 10-cell-recipients on day 24 after engraftment. The total number of GCs per section was comparable in the two groups (Fig. 2 *H*–*J*), yet HkiL cell progeny (CD45.1^+^) were consistently found in PNA^+^ GC structures of DOC-LAV-infected 10-cell-recipients but were virtually absent from GCs of DOC-infected 10-cell-recipients (Fig. 2 *H*, *I*, and *K*). These observations on clonal attrition of high- but not low-affinity B cells contrasted with earlier studies in the context of protein vaccination, showing that high-affinity B cells mounted equal if not stronger responses than their low-affinity counterpart (38, 61).

### B Cell Attrition Occurs in the Presence of Excessive Amounts of Viral Antigen but Independently of IFN-I Signaling.

**Fig. 3.**
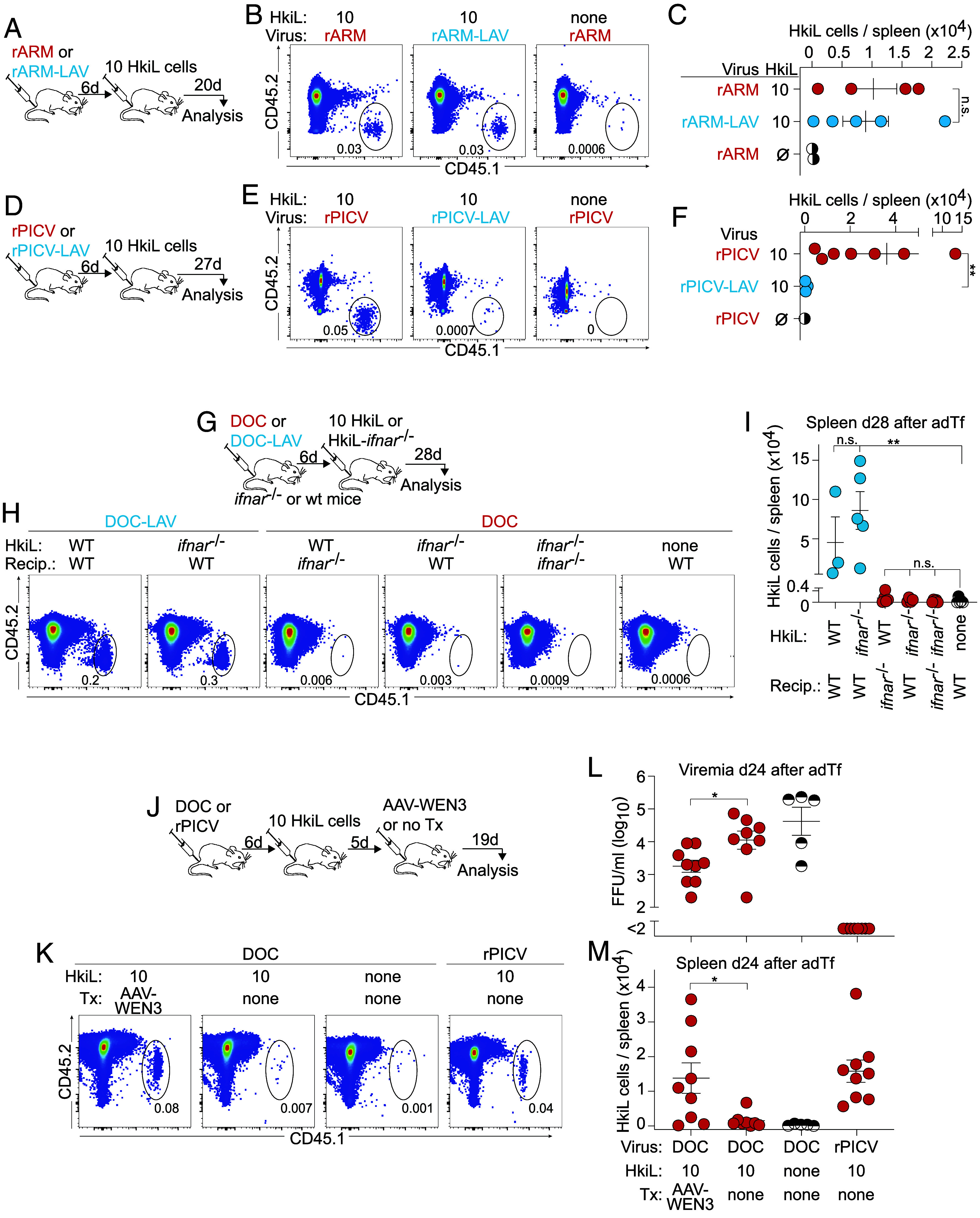
B cell attrition occurs in the presence of excessive amounts of viral antigen but independently of IFN-I signaling. (*A*–*C*) We infected mice with rARM or rARM-LAV on d-6 and on d0 engrafted 10 HkiL cells or none (noTf) (*A*). HkiL progeny in the spleen were analyzed and enumerated on d20 (*B* and *C*). Representative FACS plots (*B*; n = 4 mice except n = 2 mice in noTf group) pregated on live lymphocytes (*SI Appendix,* Fig. S1*A*). The cells’ expression of B220 and CD138, and the GL7/CD38 profile of the B220^+^CD138^−^ subset are reported in *SI Appendix,* Fig. S3*A*. (*D*–*F*) We infected mice with rPIC or rPIC-LAV on d-6 and on d0 engrafted 10 HkiL cells or none (noTF) (*D*). HkiL progeny in the spleen were analyzed and enumerated on d27 (*E* and *F*). Representative FACS plots (*E*; n = 4 mice for rPIC, n = 3 mice for rPICV-LAV group, n = 1 for noTF) pregated on live lymphocytes (*SI Appendix,* Fig. S1*A*). The cells’ expression of B220 and CD138, and the GL7/CD38 profile of the B220^+^CD138^−^ subset are reported in *SI Appendix,* Fig. S3*C*. (*G*–*I*) We infected *ifnar*^−/−^ and WT control recipients with DOC or DOC-LAV on d-6. On d0, we engrafted them with either 10 HkiL cells or 10 HkiL-*ifnar*^−/−^ cells or left them without cell Tf (“none”, noTF) in the combinations indicated in (*H* and *I*). HkiL and HkiL-*ifnar*^−/−^ progeny (CD45.1^+^) were analyzed on d28 (*G* and *I*). Representative FACS plots [n = 5 except HkiL into DOC-LAV-infected WT mice (n = 3) and HkiL-*ifnar*^−/−^ into DOC-infected wt mice (n = 4)], pregated on live lymphocyte are shown in (*H*) and CD45.1^+^ progeny were enumerated (*I*). The cells’ expression of CD19 and CD138, and the GL7/CD38 profile of the CD19^+^CD138^−^ subset are reported in *SI Appendix,* Fig. S3*D*. (*J*−*M*) We infected mice with DOC or rPIC on d-6 and on d0 engrafted 10 HkiL cells or none (noTF) as indicated in the chart to (*M*). On d5, we administered AAV-WEN3 to one DOC-infected group. HkiL progeny in the spleen were analyzed on d24 (*J*). Representative FACS plots (*K*; n = 9 AAV-WEN3-treated HkiL recipients, n = 8 recipients without AAV-WEN3, n = 9 rPICV-immunized HkiL recipients, n = 5 noTF controls) pregated on live lymphocytes. Viremia (*L*) and total splenic HkiL progeny (*M*) on d24. The cells’ expression of CD19 and CD138, and the GL7/CD38 profile of the CD19^+^CD138^−^ subset are reported in *SI Appendix,* Fig. S3*F*. Numbers in FACS plots indicate the percentage of gated cells (*B*, *E*, *H*, and *K*) as mean of the group. Symbols in (*C*, *F*, *I*, *L*, and *M*) show individual mice with the mean ± SEM indicated. Student’s *t* tests of log-converted values were performed in (*C* and *F*). Values in (*I*) were log-converted and analyzed by one-way ANOVA with Dunnett’s posttest to identify significant differences from the noTF group. Student’s *t* tests were performed in (*L* and *M*). Data in (*A*−*I*) show one out of two similar experiments, (*L* and *M*) show combined data from two experiments. *P < 0.05; **P < 0.01; n.s.: not statistically significant.

To test the hypothesis that attrition was inherently linked to persistent infection, we assessed the response of HkiL cells to acute infection and vectored vaccination. We infected mice with either one of two engineered variants of the nonpersisting LCMV strain Armstrong, expressing either WE-GP or its low-affinity N119S-mutant (rARM; rARM-LAV), engrafted them with 10 HkiL cells, and analyzed these cells’ progeny 20 d later (Fig. 3*A*). Comparable numbers of HkiL cell progeny were found in both infection settings, indicating that attrition was not observed in the context of acute, nonpersisting infection (Fig. 3 *B* and *C* and *SI Appendix,* Fig. S3*A*). To corroborate and extend these conclusions, we engineered Pichinde virus, which replicates poorly in mice (*SI Appendix,* Fig. S3*B*) but shows utility as a viral vaccine vector (62), to express either WE-GP or the low-affinity N119S variant (rPICV; rPICV-LAV) instead of its own GP. When infected with rPICV, 10-cell-recipients formed a substantial population of HkiL cell progeny, whereas in rPICV-LAV-infected recipients, HkiL cell progeny were significantly less abundant (Fig. 3 *D–F* and *SI Appendix,* Fig. S3*C*). These observations demonstrated that 10 engrafted HkiL cells were perfectly able to mount sustainable responses to high-affinity WE-GP, provided the latter was presented in the context of acute infection or vaccination.

Attrition in chronically infected mice was observed at around 2 wk after HkiL cell Tf into preinfected recipients (compare Fig. 1 *F*−*J* and below), which differs profoundly from *decimation* (15–17). The latter occurs concomitantly with the peak of the IFN-I response during the first 3 d after LCMV infection and is largely avoided when transferring specific B cells a few days later, as it was done in the present set of experiments (15–18). Nevertheless, we set out to formally address the possibility that attrition was due to persisting low-level IFN-I signaling in chronic LCMV infection (63). IFN-I receptor-sufficient and -deficient HkiL cells (HkiL; HkiL-*ifnar*^−/−^) expanded comparably when engrafted in DOC-LAV-infected recipients (Fig. 3 *G*–*I* and *SI Appendix,* Fig. S3 *D* and *E*), allowing us to test whether B cell–intrinsic and/or –extrinsic IFN-I signaling might contribute to B cell attrition in the context of DOC infection. By transferring either 10 HkiL-*ifnar*^−/−^ cells into wt recipients, 10 HkiL cells into *ifnar*^−/−^ recipients, or 10 HkiL-*ifnar*^−/−^ cells into *ifnar*^−/−^ recipients, we found that neither B cell–intrinsic IFNAR deficiency, nor recipient IFNAR deficiency, or a combination thereof prevented attrition.

We hypothesized that B cell attrition in chronic infection may be due, at least in part, to the incessant exposure to copious amounts of cognate high-affinity antigen. To address this possibility, we sought to reduce the antigenic stimulation of HkiL cells by masking their cognate antigen by so-called antibody feedback (64). 5 d after HkiL cell engraftment, we treated DOC-infected 10-cell-recipients intramuscularly with an adeno-associated viral vector, a somatic gene therapy, for the continuous high-level production of the DOC-nAb WEN3 (AAV-WEN3; Fig. 3*J*) (46, 65). WEN3 competes with KL25 for the same epitope (66), but typical KL25 escape mutations in the viral GP such as N119S in DOC-LAV do not affect WEN3 binding or neutralization (46). When analyzed 24 d after HkiL cell Tf, AAV-WEN3 treated 10-cell-recipients exhibited significantly lower viral loads than untreated controls (Fig. 3*L*). Unlike in the latter, where HkiL cells had undergone attrition, HkiL progeny populations were preserved in the majority of AAV-WEN3-treated mice, reaching levels comparable to those of an rPICV-vaccinated reference group (Fig. 3 *K* and *M* and *SI Appendix,* Fig. S3*F*). These results suggested that the long-term exposure to very high amounts of high-affinity antigen rather than IFN-I signaling accounted for B cell attrition in chronic viral infection.

### HkiL Cell Populations Destined to Attrition Mount a Transient ASC Burst and Exhibit an Altered GC Phenotype.

**Fig. 4.**
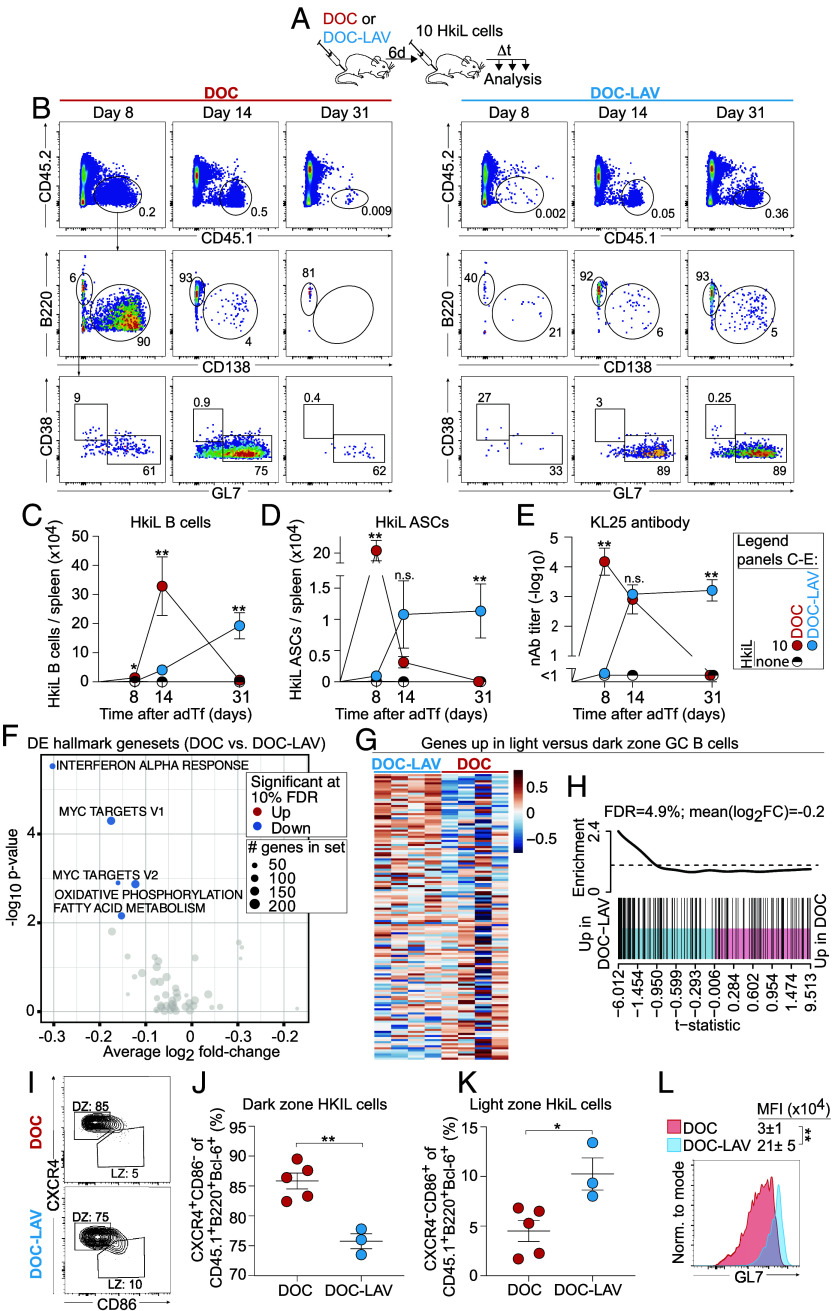
On their path to attrition, high-affinity B cells pass through an ASC burst and a subsequent DZ–biased GC stage. *A–E*: We infected mice with DOC or DOC-LAV on d-6 and on d0 engrafted either 10 HkiL cells or none (none, noTF) (*A*). HkiL progeny in the spleen were analyzed on d8, d14, and d31. Representative FACS plots [*B*; n(d8, d14, d31) = 4, 4, and 3 mice for DOC; n(d8, d14, d31) = 3, 3, and 5 mice for DOC-LAV; n(d8, d14, d31)=1, 1, and 3 mice for noTf], pregated on live lymphocytes (*SI Appendix,* Fig. S1*A*). Splenic HkiL progeny (CD45.1^+^, B: *Top*) were analyzed for expression of B220 and CD138 (*B*: center), and their B220^+^CD138^−^ subset was analyzed for GL7 and CD38 expression (*B*: *Bottom*). CD45.1^+^B220^+^CD138^-^ HkiL progeny of B cell phenotype and CD45.1^+^B220^−^CD138^+^ HkiL progeny of ASC phenotype were enumerated (*C* and *D*), and (HkiL cell-derived) nAb titers in serum (*E*) were determined over time. (*F* and *G*) In an experiment using green fluorescent protein- (GFP-) transgenic HkiL cells and conducted as in (*A*), we sorted on d14 live CD45.1^+^B220^+^CD138^−^GFP^+^ HkiL B cell progeny (*SI Appendix,* Fig. S4*A*). Total RNA of the sorted cells was processed for bulk RNAseq (*F*–*H*). Differentially expressed hallmark gene sets with a false discovery rate < 0.1 are shown in (*F*). Heat maps display the expression of genes reported to be upregulated in LZ as compared to DZ GC B cells (68) (*SI Appendix,* Fig. S4*C*). Each column of the heatmap represents an individual mouse (n = 4 per group). Pair-wise self-contained gene set testing (*H*). (*I*–*L*) In an experiment conducted as in (*A*), we analyzed HkiL progeny in the spleen on d17. Representative FACS plots (n = 5 for DOC, n = 3 for DOC-LAV), pregated on live CD45.1^+^B220^+^Bcl6^+^ HkiL cells (*SI Appendix,* Fig. S4*D*) showing their LZ (CD86^+^CXCR4^−^) and DZ (CD86^−^CXCR4^+^) distribution (*I*), proportional repartition into these zones (*J* and *K*) and GL7 expression profile (*L*). Representative histogram plots pregated on live CD45.1^+^CD19^+^Bcl6^+^ HkiL GC B cells (*SI Appendix,* Fig. S4*D*). Numbers in FACS plots indicate the percentage of gated cells as the group mean (*B* and *I*). Symbols and error bars in (*C–E*) show the mean ± SEM. For statistical analysis, cell numbers in (*C* and *D*) were log-converted and samples without any detectable HkiL cells were assigned the highest count recorded in the noTf group. For each time point the values of DOC- and DOC-LAV-infected mice were compared by unpaired Student’s *t* tests with Bonferroni correction. Two-way ANOVA with Bonferroni’s multiple comparison test was performed in (*E*) and for each time point significant differences between DOC- and DOC-LAV-infected mice are reported. Unpaired Student’s *t* tests were performed in *J*–*L*. Data in (*B*–*E* and *I*–*L*) show one representative out of two similar experiments. *P < 0.05; **P < 0.01; n.s.: not statistically significant.

To investigate the cellular differentiation processes associated with attrition, we compared HkiL cell populations in DOC- and DOC-LAV-infected 10-cell-recipients over time (Fig. 4*A*). By day 8 after engraftment, HkiL cells responding to DOC infection had given rise to a massive burst of CD138^+^B220^low/int^ ASCs, which contracted by day 14 and reached detection limits on day 31 (Fig. 4 *B* and *D*). Serum KL25 antibody titers followed these kinetics (Fig. 4*E*). In contrast, B220^hi^ B cell phenotype HkiL progeny in the same recipients increased in numbers between day 8 and day 14, exhibited predominantly a GC phenotype at both time points and disappeared thereafter (Fig. 4*C*). These kinetics differed from HkiL cells responding to DOC-LAV infection. B cell phenotype HkiL progeny numbers increased continuously from day 8 to day 31, whereas ASC numbers and antibody titers reached their maximum on day 14 and remained constant thereafter. On day 14 after engraftment, HkiL cell progeny with a GC phenotype were present in both DOC- and DOC-LAV-infected 10-cell-recipients, offering an opportunity for a comparative analysis of transcriptional signatures associated with attrition. We purified HkiL GC B cells from DOC- and DOC-LAV-viremic recipients by FACS on day 14 (*SI Appendix,* Fig. S4 *A* and *B*) and processed them for bulk RNAseq (67). A gene set enrichment analysis on the MSigDB hallmark pathways revealed that target genes of c-Myc, which is downregulated in GC DZ cells (68), were expressed at lower levels when HkiL GC B cells responded to DOC infection as compared to DOC-LAV infection (Fig. 4*F*). Consistent therewith, the comparison to a published gene set for GC DZ vs. LZ differentiation (68) indicated that HkiL GC B cells in DOC-infected animals exhibited a more pronounced DZ signature than their counterpart in DOC-LAV-infected mice (Fig. 4 *G* and *H* and *SI Appendix*, Fig. S4*C*). This DZ bias of Bcl6^+^B220^+^ HkiL GC B cells from DOC-infected animals was also reflected in an increased proportion of CXCR4^+^CD86^−^ DZ) cells and a reduced fraction of CXCR4^−^CD86^+^ LZ cells in flow cytometry (Fig. 4 *I*–*K* and *SI Appendix*, Fig. S4*D*). Efficient GC reactions require B cells to attenuate their BCR signaling and the failure to do so can result in a DZ bias (69, 70). One pathway to attenuated BCR signaling comprises the remodeling of the surface glycan composition on GC B cells (69), which can be detected by means of the widely used GL7 antibody. Interestingly, Bcl6^+^B220^+^ HkiL GC B cells from DOC-infected recipients exhibited lower levels of GL7 than those from DOC-LAV-infected controls (Fig. 4*L*). Taken together, these findings suggested that persistent viral infection resulted in phenotypic alterations that preferentially affected high-affinity GC B cells.

### Attrition of High-Affinity B Cells in Chronic Viral Infection Is Blimp-1 Dependent.

**Fig. 5.**
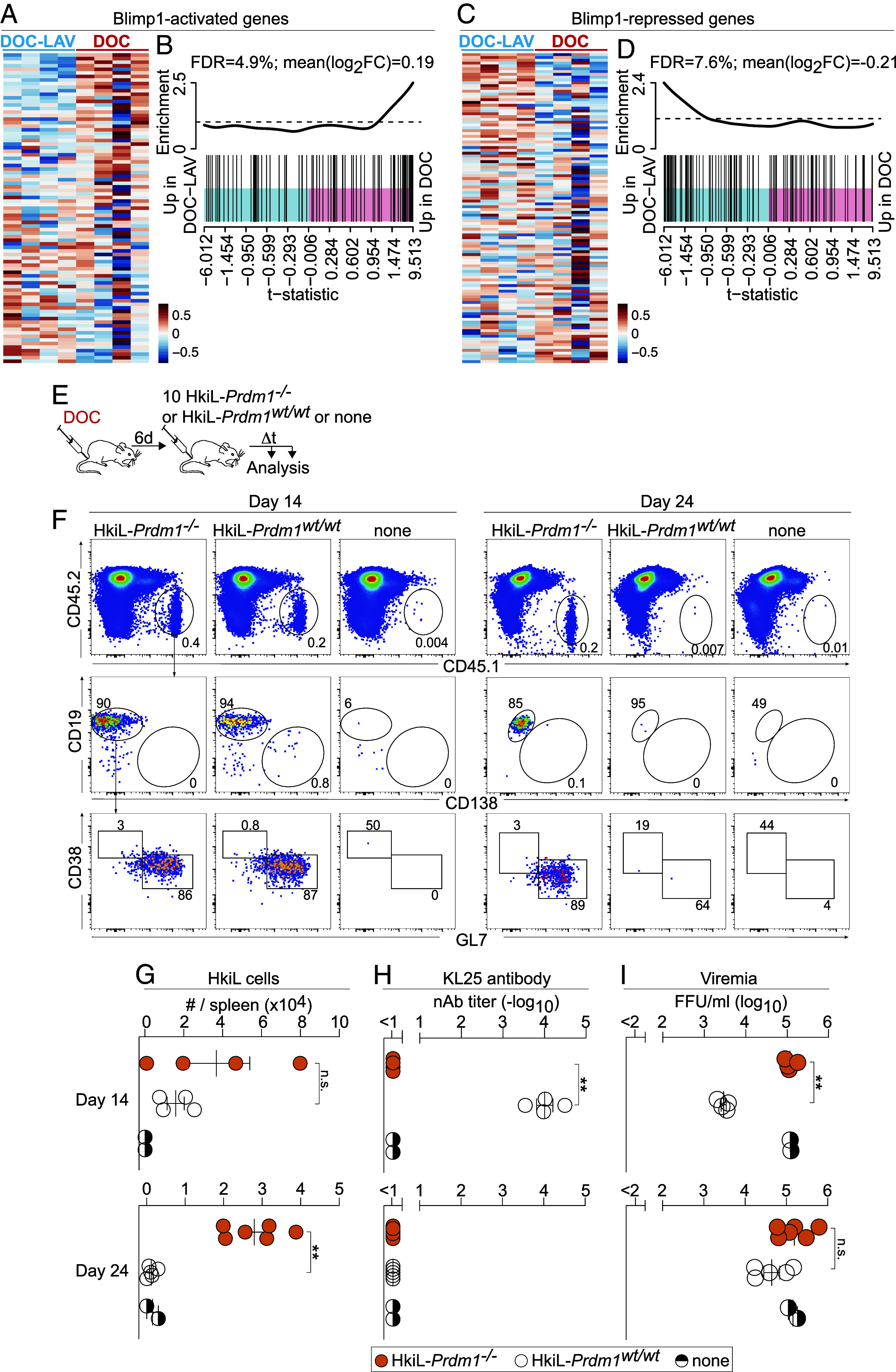
Attrition of high-affinity B cells in chronic viral infection is Blimp-1 dependent. (*A–D*) The datasets from Fig. 4 *F*–*H* were analyzed for Blimp-1-activated (*A* and *B*) and Blimp-1-repressed genes (*C* and *D*) (71). See also *SI Appendix,* Fig. S5 *A* and *B*. Each column of the heatmaps in (*A* and *C*) represents an individual mouse (n = 4 per group). Pair-wise self-contained gene set testing is shown in (*B* and *D*). (*E*–*I*) We infected mice with DOC on d-6; on d0, we engrafted either 10 HkiL-*Prdm1*^−/−^ or 10 HkiL-*Prdm1*^wt/wt^ cells or none (noTF) and we analyzed these cells’ progeny in the spleen on d14 and on d24. Representative FACS plots (*F*; d14: 4 HkiL-*Prdm1*^−/−^ recipients, 4 HkiL-*Prdm1*^wt/wt^ recipients, 2 noTf; d24: 6 HkiL-*Prdm1*^−/−^ recipients, 5 HkiL-*Prdm1*^wt/wt^ recipients, 2 noTf), pregated on live lymphocytes (*SI Appendix,* Fig. S1*A*). HkiL progeny (CD45.1^+^, *Top*) were analyzed for expression of CD19 and CD138 (center), and their CD19^+^CD138−^−^ subset was analyzed for GL7 and CD38 expression (*Bottom*). HkiL cell progeny (*G*), KL25 serum antibody (*H*), and viremia (*I*) were determined on d14 and d24. Numbers in FACS plots indicate the percentage of gated cells as the group mean (*F*). Values of HkiL-*Prdm1*^−/−^ and HkiL-*Prdm1*^wt/wt^ recipients in *G*–*I* were compared by unpaired Student’s *t* tests. Results in (*E*–*I*) show one representative out of two experiments. **P < 0.01; n.s.: not statistically significant.

In addition to the above transcriptional alterations, HkiL GC B cells from DOC-infected mice exhibited a more pronounced Blimp-1 signature than those responding to DOC-LAV. More specifically, genes known to be activated by the transcription factor Blimp-1 (71) (encoded by the *Prdm1* gene) were higher in DOC- than in DOC-LAV-infected mice, whereas Blimp-1-repressed genes exhibited the opposite pattern (Fig. 5 *A*–*D* and *SI Appendix,* Fig. S5 *A* and *B*).

Blimp-1 is a master transcription factor of plasma cell differentiation and can also play an important role in the transcriptional regulation of GC B cells (72–76), prompting us to test whether Blimp-1 expression in B cells was required for attrition. Blimp-1 deficiency is embryonically lethal, but fetal liver cells from animals homozygous for a functional null allele of Prdm1 (encoding for Blimp-1; *Prdm1*^−/−^ mice) can be used to reconstitute irradiated recipients and give rise to mature B cells (74). Following this strategy, we generated donors of either Blimp-1-deficient or -sufficient HkiL cells (HkiL-*Prdm1*^−/−^, HkiL-*Prdm1*^wt/wt^) from which we transferred 10 cells into DOC-infected recipients. When analyzed 14 d later, progeny of both types of cells were readily detectable (Fig. 5 *E*–*G*). As expected, HkiL-*Prdm1*^−/−^ progeny were virtually devoid of CD138^+^B220^low/int^ ASCs, they failed to produce detectable amounts of KL25 serum antibody and, presumably as a consequence thereof, the corresponding recipients had higher viral loads than those engrafted with HkiL-*Prdm1*^wt/wt^ cells (Fig. 5 *F*, *H*, and *I*). When recipients were analyzed 24 d after engraftment, HkiL-*Prdm1*^wt/wt^ cells had undergone attrition, as expected (Fig. 5 *F* and *G*). In contrast, HkiL-*Prdm1*^−/−^ progeny persisted in DOC-infected recipients in numbers comparable to those determined on day 14, thus offering no evidence of attrition. Unlike in the context of chronic DOC infection, Blimp-1-deficient and -sufficient HkiL cells expanded and persisted in similar numbers when triggered by rPICV vaccination (*SI Appendix,* Fig. S5 *C*–*F*). Hence, Blimp-1 deficiency prevented the attrition of high-affinity B cells in chronic viral infection but did not afford a clear advantage to B cells responding to vaccination. Taken together, the resistance of Blimp-1-deficient B cells to attrition suggested the latter was the consequence of a cell-intrinsic reprogramming of GC B cells in the context of chronic viral infection.

## Discussion

The present findings in the context of chronic LCMV infection suggest that antiviral B cells with a high-affinity receptor mount a transient response and are eliminated from detectable B cell compartments by attrition unless they can clear viremia or drive viral mutational escape. The naïve precursors of LCMV- and HIV-nAb-producing B cells in mice and humans, respectively, are, however, mostly of low to intermediate affinity, and substantial hypermutation is required for these cells to reach affinity ranges that confer neutralizing capacity (18, 30, 31). Our observations predict on the one hand that the average nAb-producing B cell precursor will efficiently expand and affinity-mature in chronically infected individuals. On the other hand, our data suggest that each clone’s highest-affinity progeny will be pruned by attrition, thus continuously ridding the antiviral B cell response of its most proficient output.

Exogenously supplied as well as endogenously produced antibodies can modulate antigen- and/or epitope-specific GC B cell responses by so-called “antibody feedback” i.e., by blocking B cell access to antigen (64). While commonly known for its negative regulatory effects in the context of vaccination i.e. under conditions of limited antigen availability, the present findings raise the possibility that attenuation of BCR signaling by antibody feedback may benefit GC B cell responses under conditions of chronic high-level viremia. Accordingly, the protective effect of AAV-WEN3 therapy against HkiL cell attrition in DOC infection may help explain the observation that bnAb treatment of HIV-viremic individuals augmented their endogenous HIV-nAb response (77) and, analogously, that LCMV-nAb therapy improved polyclonal virus-specific GC B cells responses in chronically infected mice (65). The molecular mechanism whereby AAV-WEN3 therapy attenuates HkiL cell attrition requires, however, further investigation. Besides attenuation of BCR signaling as discussed above, the viral load reduction afforded by the passively administered nAb may exert additional beneficial effects on HkiL cells e.g. by reducing detrimental consequences of virus-induced inflammation (15–17, 63). Also, the mechanism whereby Blimp-1 deficiency protects Hkil cells from attrition warrants further investigation. Prevention of terminal plasmablast differentiation seems one likely hypothesis. Blimp1-mediated downregulation of Bcl6 and upregulation of IRF4 (76) may have promoted attrition as suggested by the massive early ASC burst typical of the HkiL cell response to DOC. Not mutually exclusively, Blimp-1 deficiency may allow HkiL B cells in persistently infected mice to proliferate more and/or to die at a lower rate, since Blimp-1 negatively regulates cell-cycle progression and c-Myc expression by GC B cells (76, 78). In light of repressed c-Myc transcriptional signatures in HkiL cells destined to attrition, it seems plausible that Blimp1 deficiency counteracted attrition by enabling the cells to maintain critical c-Myc levels and to more efficiently progress in cell cycle.

Of note in this context, ASC-biased B cell compartments represent a long-standing observation in HIV-viremic individuals and have been associated with subversion of humoral immunity (11). While preferential ASC differentiation of high-affinity B cells serves presumably to ascertain the effectiveness of the serum antibody responses in the context of acute infection and vaccination (73, 79), we propose that in chronic viral infection, the same may culminate in attrition, thus promoting humoral immune subversion.

Our study with its experimental set-up has clear limitations. LCMV infection of mice represents an imperfect surrogate of human persistent viral diseases despite having contributed concepts of broad utility such as the exhaustion of CD8 T cells in persistent viral infection, their reinvigoration by PD-1-targeted therapy and mutational escape from CD8 T cell control (6, 7, 80). Moreover, our study relied on the adoptive Tf of a monoclonal population of receptor knock-in B cells. While representing the only experimental approach whereby to control key parameters such as the affinity of antiviral B cells and the timing of their recruitment into the immune response, the possibility remains that other B cell clones in other infection settings would respond differently. Observations with adoptively transferred HBV-specific B cells in a mouse model of persistent HBV infection indicate, however, that a plasmablast burst followed by the disappearance of virus-specific B cells from the GC is not unique to HkiL cells in chronic LCMV infection (81). Last but not least, the disproportionate decline of KL25 antibody titers in 10-cell recipients suggested attrition was not restricted to specific tissues, but our flow cytometric analyses were limited to spleen, inguinal lymph nodes, and bone marrow and thus cannot exclude the possibility that HkiL cell progeny persisted in other anatomical locations.

Taken together, this study describes the affinity-dependent clonal attrition of specific B cells during viral persistence, suggesting the chronic infection context perturbs affinity hierarchies of antiviral B cell responses. A refined understanding of humoral immune subversion by persisting viruses with molecular insights into the underlying mechanisms may open broad avenues for therapeutic interventions aimed at improving humoral immune control of chronic microbial diseases.

## Materials and Methods

Materials and Methods are provided as a part of the *SI Appendix* and detail mice and animal experiments, generation of HkiL-*Prdm1^−^*^/−^ and HkiL-*Prdm1*^wt/wt^ control B cell donors, TaqMan PCR-based genotyping of HkiL-*Prdm1^−/−^* and HkiL-*Prdm1*^wt/wt^ embryos, cells, viruses, virus titration and infection of mice, adeno-associated viral vectors and their administration to mice, adoptive B cell Tf, flow cytometry, determination of GP1-binding and LCMV-nAb concentrations in mouse serum, viral sequence determination, FACS sorting and bulk RNA sequencing of HkiL cells, analysis of bulk RNA sequencing data, immunohistochemistry, and image analysis as well as statistical analysis. Animal experiments were performed at the University of Basel in accordance with the Swiss Law for animal protection and with authorization from the Veterinary Office Basel Stadt.

## Supplementary Material

Appendix 01 (PDF)

## Data Availability

scRNAseq data are deposited at GEO under GSE296780 (67). Raw data of the experimental results reported in this study will be deposited with Zenodo and will be publicly available as of the date of publication under https://doi.org/10.5281/zenodo.15600975 (49, 50).
