# Supplementary material for: Chronic infection perturbs the affinity hierarchy of antiviral B cells
Source: Proc Natl Acad Sci U S A. 2026 Mar 31;123(14):e2532422123. doi: 10.1073/pnas.2532422123 (PMC13056152; doi:10.1073/pnas.2532422123)
Supplement: Supplementary file 1 — Appendix 01 (PDF) [file pnas.2532422123.sapp.pdf]

Materials and Methods

Mice and animal experiments

C57BL/6J wildtype mice were originally purchased from Charles River and were bred locally for experiments. HkiL mice carrying both a B cell receptor (BCR) heavy and light chain V(D)J knock-in originating from the LCMV-neutralizing GP-1-specific monoclonal antibody (mAb) KL25 have been described (1). TgL mice express a transgenic version of the KL25 light chain and were used as recipients for HkiL B cells to avoid anti-idiotypic rejection (1-4). B cell-deficient JHT mice (5) and interferon receptor deficient mice (*ifnar*$^{-/-}$)(6) were obtained from the Swiss Immunological Mouse Repository (SwImMR). HkiL mice were crossed to *ifnar*$^{-/-}$ mice to obtain HkiL-*ifnar*$^{-/-}$ cell donors (CD45.1^{+}). As recipients of HkiL-*ifnar*$^{-/-}$ and control HkiL B cells in the respective experiments we used *ifnar*$^{-/-}$ mice that were intercrossed with TgL mice (for simplicity referred to as *ifnar*$^{-/-}$ mice in the manuscript's text). HkiL mice were intercrossed with *Prdm1*$^{-/wt}$ mice (7) with a GFP knock-in disrupting the *Prdm1* locus, and separately with UBI-GFP mice (C57BL/6-Tg(UBC-GFP)30Scha/J; Jax #004353) to obtain HkiL-*Prdm1*$^{gfp/wt}$ mice and HkiL-GFP mice, respectively (both CD45.1^{+}). HkiL, HkiL-GFP and HkiL-*Prdm1*$^{gfp/wt}$ mice were kept on a RAG2-deficient background (1). To prevent rejection of GFP-expressing HkiL-*Prdm1*$^{-/-}$ B cells (see below) and control HkiL cells in the respective experiments we used TgL mice with liver-specific expression of EYFP. For this, TgL mice were intercrossed with Albumin-Cre (B6.Cg-Speer6-ps1Tg(Alb-cre)21Mgn/J; Jax #003574) and R26-stop-EYFP mice (B6.129X1-Gt(ROSA)26Sortm1(EYFP)Cos/J; Jax #006148) . All mouse lines were on a C57BL/6J background and were bred at the ETH Phenomics Center (EPIC) of the Swiss Federal Institute of Technology Zurich or at the University of Basel mouse breeding facility under specific pathogen-free (SPF) conditions. Experiments were performed at the University of Basel in accordance with the Swiss Law for animal protection and with authorization from the Veterinary Office Basel Stadt. Adult animals from both genders were used to reduce the number of animals bred for research purposes. Within each experiment the animals were age- and sex-matched. Experiments were not conducted in a blinded fashion.

Generation of HkiL-*Prdm1*$^{-/-}$ and HkiL-*Prdm1*$^{wt/wt}$ control B cell donors

HkiL-*Prdm1*$^{-/wt}$ mice homozygous for the B cell receptor (BCR) heavy and light chain V(D)J knock-ins were mated with *Prdm1*$^{-/wt}$ mice and 48 hours later the males were removed from the breeding cages. Fourteen days after mating the females were sacrifices, embryos were removed, their livers were extracted, placed in DPBS supplemented with 3% FCS and kept on ice. A piece of each embryo's front paw was processed for DNA extraction using the QIAmp Fast DNA tissue kit (Quiagen). Upon identification of *Prdm1*$^{-/-}$ and *Prdm1*$^{wt/wt}$ control embryos by TaqMan PCR (see below), the respective liver samples were filtered through a 70 μm cells strainer and combined with JHT bone marrow for intravenous implantation into lethally irradiated (11 Gy one day beforehand) C57BL/6J recipients. Reconstitution of the recipients with HkiL-*Prdm1*$^{-/-}$ or HkiL-*Prdm1*$^{wt/wt}$ control B cells (CD45.1^{+}) of fetal liver cell origin was verified in the respective recipient's blood ≥30 days later, and successfully reconstituted mice were used as B cell donors for adoptive transfer experiments in Fig. 5E-I. For the experiment reported in Fig. S5C-F splenic B cells from a non-chimeric HkiL mouse were used as HkiL-*Prdm1*$^{wt/wt}$ control cells.

TaqMan PCR-based genotyping of HkiL-*Prdm1*$^{-/-}$ and HkiL-*Prdm1*$^{wt/wt}$ embryos

To identify HkiL-*Prdm1*$^{-/-}$ and HkiL-*Prdm1*$^{wt/wt}$ control embryos and to differentiate them from HkiL-*Prdm1*$^{-/wt}$ animal we performed TaqMan PCR analyses on DNA extracted from embryonic biopsies as described above. In brief, the extracted DNA was quantified by optical density measurements and the concentration of samples was adjusted to 50 ng/μl. The samples were then analyzed in duplicates on a StepOnePlus Real-Time PCR System 96-well device (Applied Biosystems) using the following primer-probe sets. The unmodified *Prdm1* locus was amplified using primers 5'-CTTGGCTGCCAGGCAGTGC-3' and 5'-GCCAGGTATGTACAATGCAGATGC-3' with a probe 5'-FAM-TTGAGCCATAGGAGACC-BHQ1-3'. The *Prdm1*-ko locus was detected by amplification of the inserted GFP sequence using primers 5'-CTCGTGACCACCTTGACCTA-3' and 5'-GAAGTCGTGCTGCTTCATGT-3' with the probe 5'-FAM-CGGACGAAGCACTGCACGCCG-BHQ1-3'. To normalize for DNA content in the reactions we used ApoB-binding primers 5'-CACGTGGGCTCCAGCATT-3' and 5'-TCACCAGTCATTTCTGCCTTTG -3' with the probe 5'-FAM-CCAATGGTCGGGCACTGCTCAA-BHQ1-3'.

Cells, viruses, virus titration and infection of mice

NIH 3T3 cells (CRL-1658) and MDCK cells (CCL34) were purchased from ATCC. BHK21 cells (85011433) and VeroE6 cells were both from ECACC (# 85020206). All cells were cultured at 37 °C in an atmosphere of 5 % CO_2. They were regularly tested for mycoplasma and confirmed negative. The genetically engineered LCMV clone 13-based rCl13/WE and the Armstrong-based rARM/WE have been described (8, 9). rARM/WE-LAV was generated from cDNA as previously described (10). The wildtype LCMV strain Docile (11) (DOC) was originally obtained from Rolf Zinkernagel. A cDNA rescue system for DOC was built using as a basis a published set of plasmids (12) that were generously provided by Dimitrios Moskophidis. Modifications to the published viral sequence (12) were made as outlined in Fig. S2B. cDNA-derived DOC of wildtype sequence as well as DOC with the N119S point mutation in its GP (DOC-LAV) were generated following established procedures (10) and were used throughout the study except for the wildtype DOC isolate used in the experiment in Fig. S2B as indicated. Pichinde- (PICV-) based viruses expressing instead of their natural envelope glycoprotein the LCMV strain WE glycoprotein (rPICV) or its N119S-mutant (rPICV-LAV) were engineered as described (13). Clone 13- and Armstrong-based viruses were propagated on BHK-21 cells, DOC-based viruses on MDCK cells. For infection of mice rCl13/WE, DOC and DOC-LAV were administered intravenously at a dose of ≥2x10e6 focus-forming units (FFU), rARM/WE and rARM/WE-LAV were administered intraperitoneally at a dose of 200 FFU. rPICV and rPICV-LAV were administered intravenously at a dose of 10e6 FFU. For the determination of viremia one drop of blood was collected into 950 μl of BSS supplemented with 1 IE/ml heparin (Na-Heparin, Brown). Infectious titers in viral stocks and blood samples of animals infected with LCM viruses or immunized with PICV-based viruses were determined by immunofocus assays on NIH 3T3 cells as described (13, 14).

Adeno-associated viral vectors and their administration to mice

An adeno-associated viral (AAV) vector genome expressing the LCMV-neutralizing mAb WEN3 (2, 15, 16) (AAV-WEN3) was designed and generated following established strategies and procedures (17, 18). AAV vector

particles with an AAV8 capsid were produced and titrated by the Viral Vector Facility (VVF) of the University of Zurich, Switzerland. AAV-WEN3 was administered to mice at a dose of 10e11 vector particles (v.p.) in a total volume of 40 µl into both thigh muscles.

Adoptive B cell transfer

For adoptive transfer of B cells from HkiL and HkiL-GFP mice as well as from HkiL-*Prdm1*$^{-/-}$ and HkiL-*Prdm1*$^{wt/wt}$ chimeras, splenic single cell suspensions were generated by mechanical disruption and B cells were purified using the EasySep mouse B cell isolation kit (STEMCELL, #19854) following the manufacturer's instructions. The cells were adoptively transferred into the tail vein in either BSS or RPMI. Assuming a splenic take of ~5% ((19-21); Fig. S1), we administered 200 purified B cells to engraft 10 cells into the spleen of recipients (10-cell-recipients), 600 B cells to generate 30-cell recipients, 2,000 B cells for 100-cell recipients and 20,000 cells for 1000-cell-recipients. The group of mice labeled as "100-cell" recipients in Fig. 1K-O summarizes two groups of mice given 2000 and 5000 cells, respectively, to engraft 100 or 250 HkiL cells.

Flow cytometry

To obtain single cell suspension, spleens were mechanically disrupted using a metal grid and syringe plunger. The cells were then resuspended in RPMI (Sigma, R2405) supplemented with 20mM HEPES, 5mM $MgCl_2$, 1mM NaPyruvate, MEM non-essential amino acids, 5% FCS, Penicillin / Streptomycin and 0.05 mM β-mercaptoethanol. Media and buffers were adjusted to mouse osmolarity using NaCl (22). Lymph nodes were mechanically disrupted using a syringe plunger and a 70 µm cells strainer and resuspended in FACS buffer (PBS supplemented with 10% Opti-MEM, 1% FCS, penicillin / streptomycin, 10 mM HEPES, 5 mM NaCl, 1 mM Na-Pyruvate, MEM non-essential amino acids, 0.05 mM β-mercaptoethanol and 1 g/l D-Glucose). Bone marrow was flushed out of tibiae using a syringe and then was processed as for lymph node samples. Prior to staining, spleen and lymph nodes suspensions were incubated for 10 min at room temperature (RT) with DNAse I (50 µg/ml; Roche, 10104159001) in FACS buffer.

Stains were performed in FACS buffer supplemented with polyclonal rat IgG and anti-mouse CD16/CD32 (2.42; BioXcell, BE0307) to block nonspecific antibody binding. When more than one brilliant violet dye was in the staining mix, a BD HorizonTM brilliant stain buffer (BD Biosciences, 566385) was added to the staining mix. Antibodies against CD45.1 (A20), CD45.2 (104), CD45R/B220 (RA3-6B2), CD138 (281-2/2PH1), CD19 (6D5 or 1D3), Bcl-6 (K112-91), CD38 (REA616 or 90), GL7 (GL7), CXCR4 (2B11 or L276F12), CD86 (GL1 or PO3), Ter119 (ter119), CD95 (JO2), the Zombie U.V. Fixable Viability Kit and the Zombie NIR Fixable Viability Kit were purchased from BD Biosciences, Miltenyi Biotec, Biolegend, and Thermofisher (Invitrogen, ebiosciences) and used for staining. Samples were acquired on BD LSRFortessa (BD Biosciences) and Aurora (Cytek) flow cytometers and were analyzed using FlowJo (BD Biosciences).

Determination of GP1-binding and LCMV-neutralizing antibody concentrations in mouse serum

For serum collection we used Multivette 600 Serum gel tubes (Sarstedt, 15.1674) following the manufacturer's instructions. For enzyme-linked immunosorbent assays (ELISA) we followed established procedures (23). In

brief, 96-well flat-bottom high-binding well plates were coated overnight at 4°C with anti-human-Fc antibody diluted in coating buffer (15mM Na_2CO_3, 35mM $NaHCO_3$ in ddH_2O, pH 9.6). The next day, the plates were blocked with 5% milk PBS-0.05% Tween-20 (PBST) for 1h at RT. The blocking buffer was flicked off and GP1-Fc fusion protein was added to the plates for 1h at RT followed by three washes with PBST. In a separate 96-U-bottom plate we prepared 3-fold serial dilutions of serum samples in PBST supplemented with 1% FCS, then we transferred the diluted serum into the prepared 96-well flat-bottom high-binding well plates, followed by a 1h incubation at RT. Plates were washed 3 times with PBST and incubated with secondary HRP-couples antibody in PBS supplemented with 1% FCS for 1h at RT. Plates were then washed 3 times with PBST and once with PBS and an ABTS color reaction mix was added consisting of 0.5 mg/ml ABTS (Thermo Fischer, 34026), 28 mM Na_2HPO_4 and 0.1 % H_2O_2 in H_2O and developed for 30 min. We stopped the color reaction by adding 1% sodium dodecyl sulfate (SDS) in ddH2O and measured optical density (O.D.) at a wave length of 405 nm using an Infinite M Plex reader (Tecan). To calculate serum concentrations of GP1-specific KL25 antibody produced by adoptively transferred HkiL cells we used KL25 monoclonal antibody standard. Naïve control serum samples were used as negative controls.

To determine neutralizing antibody titers in serum, samples from infected mice and naïve negative control sera were pre-diluted ten-fold and then three-fold serial dilutions were prepared in 96-well flat-bottom tissue culture plates using MEM supplemented with 2% FCS as diluent. Monoclonal KL25 antibody was included as a positive control. The sample-containing plates were irradiated in a UV chamber for 5 min to inactivate residual infectivity in mouse serum. Then the diluted serum samples were incubated with an equal volume of medium containing approximately 1000 infectious units of green fluorescent protein-expressing replication-deficient vesicular stomatitis virus (rVSV-EGFP (24), generously provided by Gert Zimmer) pseudotyped with LCMV-WE glycoprotein for 1.5h at 37°C. Next, the plates were incubated with Vero E6 cells ($3X10^4$ cells/well) for 24h at 37°C and fixed with 1% PFA. After fixation, PFA was flicked off and PBS was added until quantifying green cells using an Immunospot S6 device (C.T.L.). The 50% neutralization titer (NT_{50}) was calculated using 4-parameter non-linear regression to determine the serum dilution yielding a 50% reduction of viral infectivity. Inverted titers were log-converted and are displayed in logarithmic format.

Viral sequence determination

For viral RNA isolation and sequencing, BHK21 cells were infected with viremic mouse blood samples and supernatant was collected two days later. Viral RNA was extracted from virions using the QIAamp Viral RNA Mini Kit (QIAGEN) in accordance with manufacturer’s instructions. cDNA synthesis and PCR amplifications of the DOC GP region was performed with the OneStep RT-PCR kit (QIAGEN) according to the manufacturer’s protocol and using the primers 5’-ATGGGCCAAATTGTGACAATGTT-3’ and 5’-CAGCGTCTTTTCCAGATAGTTT-3’. Alternatively we performed reverse transcription with the SuperScript IV first strand synthesis system (Thermofisher), followed by PCR using Phusion polymerase (New England Biolabs). Primers 5’-CGCACAGTGGATCCTAGGC-3’ and 5’-GGGTGAGTTAGCTACAGGTTTC-3’ were used to amplify the DOC wildtype NP sequence. The PCR products were run on 1.5% agarose gel, amplicons of the correct size were excised,

purified using the QIAquick Gel Extraction Kit (QIAGEN) and subject to Sanger sequencing (Microsynth AG, Switzerland).

FACS-sorting and bulk RNA sequencing of HkiL cells

For bulk RNA sequencing, HkiL-GFP cells from DOC- and DOC-LAV-infected mice were bead-enriched using anti-CD45.1-PE antibody and the EasySep^{TM} PE Positive Selection Kit II (STEMCELL, 17684) according to the manufacturer's instructions. Subsequently HkiL B cells (CD45.1^{+}GFP^{+}B220^{+}CD138^{-} cells) were sorted directly into lysis buffer (TAKARA) using a FACSAriaII (Beckton Dickinson) and stored at -80°C until further use. Library preparation was performed from the direct lysis of 300 to 500 cells in 10x Lysis Buffer (Cat# 635013, Takara Bio) and Recombinant RNase Inhibitor (Cat# 2313A, Takara Bio) as described in the kit SMART-Seq Stranded Kit (Cat# 634444, Takara Bio) in conjunction with the SMARTer RNA Unique Dual Index Kit (Cat# 634452, Takara Bio) to produce ribosomal depleted libraries. The same conditions were applied for all samples with a standard time of 6 min for fragmentation, 5 cycles for PCR 1 and 14 for PCR 2, and a single final cleanup cycle. Libraries were quality-checked on the Fragment Analyzer (Advanced Analytical, Ames, IA, USA) using the High Sensitivity NGS Fragment Analysis Kit (Cat# DNF-474, Advanced Analytical) revealing excellent quality of libraries (average concentration was 16±10 nmol/L and average library size was 408±26 base pairs). Samples were pooled to equal molarity. The pool was quantified by Fluorometry using the QuantiFluor ONE dsDNA System (Cat# E4871, Promega, Madison, WI, USA) and sequenced on an Illumina NextSeq 500 instrument using the NextSeq 500 High Output Kit 75-cycles (Illumina, Cat# FC-404-1005) to produce paired-end 38nt reads (in addition: 8 bases for index 1 and 8 bases for index 2). Flow lanes were loaded at 1.8pM of pool with 1% PhiX. This Nextseq runs compiled a large number of reads (on average per sample: 42±4 millions pass-filter reads).

Analysis of bulk RNA sequencing data

Data analysis was performed by the Bioinformatics Core Facility, Department of Biomedicine, University of Basel. Read quality was assessed with the FastQC tool (version 0.11.9). Reads were mapped to the mouse genome mm39 with STAR (25) (version 2.7.10a) with default parameters, except filtering out multimapping reads with more than 10 alignment locations (outFilterMultimapNmax=10) and filtering reads without evidence in the spliced junction table (outFilterType="BySJout"). The *featureCounts (26)* function from the Rsubread package (version 2.0.6) was used to count the number of reads (5' ends) overlapping with the exons of each gene (Ensembl release 110 genes) assuming an exon union model. All subsequent analyses were performed using the R software (version 4.3.1) and Bioconductor (27) 3.18 packages. A total of 19,417 genes with CPM values above 1 in at least 3 samples (n-1 the number of biological replicates), and with a protein-coding gene biotype, were retained for the differential expression analysis. To account for complexity differences commonly observed in RNAseq libraries from low-input material, the function voomWithQualityWeights (28, 29) from the *limma* package (version 3.58.1) was used (with a cyclic loess normalization (30)) to combine observational-level with sample-specific quality weights prior to differential expression analysis comparing DOC and DOC-LAV conditions. P-values were adjusted by controlling the false discovery rate (FDR; Benjamini-Hochberg method) and genes with a FDR lower than 5% were considered

significant. Gene set enrichment analysis was performed with the function *camera (31)* from the limma package (using the default parameter value of 0.01 for the correlations of genes within gene sets) using gene sets from the mouse collections of the MSigDB Molecular Signatures Database (32) (version 2023.2), notably the hallmark gene sets collection. A collection of custom gene sets was also collected from previously published studies and datasets. We filtered out sets containing less than 10 genes, and gene sets with a FDR lower than 10% were considered significant.

Immunohistochemistry and Image analysis

For fluorescent immunohistochemistry spleen samples were fixed with HEPES-glutamic acid buffer-mediated organic solvent protection effect (HOPE, DCS Innovative, HL001R2500), subsequently embedded into paraffin and cut into 2 μm thick sections using a microtome. The CD45.1 congenic marker of HkiL cells was stained using the primary anti-mouse CD45.1-FITC antibody (A20, Invitrogen #11-0453-85) and secondary rabbit anti-FITC antibody (Invitrogen #71-1900), followed by tertiary Alexa Fluor 488 donkey anti-rabbit antibody (Life Technologies #A21206). Naïve B cells were visualized using rat anti-mouse IgD monoclonal antibody (Bioegend, clone 11-26c-2a, #405702), which had been labelled in-house with Alexa Fluor 647 (Invitrogen Labeling kit #A20186). GCs were stained with AF568-labelled peanut agglutinin (Life Technologies #L32458) and nuclear staining was performed using 4',6-diamidino-2-phenylindole (DAPI, Invitrogen). For image acquisition, slides were scanned using a Pannoramic 250 FLASH II (3DHISTECH) Digital Slide Scanner at 20x magnification.

Statistical analysis

For statistical analysis we used GraphPad Prism Software (Version 10.2.3). For the comparison of a single parameter between two groups we performed unpaired two-tailed Student's t-tests. For the comparisons of one parameter between multiple groups we performed 1-way ANOVA with Bonferroni's or Tukey's post-test. For the comparison of multiple groups against a reference control group one-way ANOVA with Dunnett's post-test was performed. Repeated measures ANOVA was performed for a pairwise comparison of time courses. To analyze repeated measurements of two or more groups we used two-way ANOVA with Tukey's or Bonferroni's post-test. $p \leq 0.05$ was considered not statistically significant (n.s.), $p<0.05$ as statistically significant (*) and $p<0.01$ as highly significant (**). Absolute cell counts, where indicated in legends, as well as antibody titers and viral loads were log-converted to obtain a near-normal distribution for statistical analysis.

Supplementary references

1. M. Florova *et al.*, Central tolerance shapes the neutralizing B cell repertoire against a persisting virus in its natural host. *Proc Natl Acad Sci U S A* **121**, e2318657121 (2024).
2. B. Fallet *et al.*, Chronic Viral Infection Promotes Efficient Germinal Center B Cell Responses. *Cell Rep* **30**, 1013-1026 e1017 (2020).
3. B. Fallet *et al.*, Interferon-driven deletion of antiviral B cells at the onset of chronic infection *Sci Immunol* **1**, eaah6817 (2016).
4. K. Narr *et al.*, Vaccine-elicited CD4 T cells prevent the deletion of antiviral B cells in chronic infection. *Proc Natl Acad Sci U S A* **118** (2021).
5. J. Chen *et al.*, Immunoglobulin gene rearrangement in B cell deficient mice generated by targeted deletion of the JH locus. *Int Immunol* **5**, 647-656 (1993).
6. S. Huang *et al.*, Immune response in mice that lack the interferon-gamma receptor. *Science* **259**, 1742-1745 (1993).
7. A. Kallies *et al.*, Plasma cell ontogeny defined by quantitative changes in blimp-1 expression. *J Exp Med* **200**, 967-977 (2004).
8. P. Penaloza-MacMaster *et al.*, Vaccine-elicited CD4 T cells induce immunopathology after chronic LCMV infection. *Science* **347**, 278-282 (2015).
9. A. Bergthaler, D. Merkler, E. Horvath, L. Bestmann, D. D. Pinschewer, Contributions of the LCMV glycoprotein and polymerase to strain-specific differences in murine liver pathogenicity. *J Gen Virol* **88**, 592-603 (2007).
10. L. Flatz, A. Bergthaler, J. C. de la Torre, D. D. Pinschewer, Recovery of an arenavirus entirely from RNA polymerase I/II-driven cDNA. *Proc Natl Acad Sci U S A* **103**, 4663-4668 (2006).
11. S. Jacobson, R. M. Friedman, C. J. Pfau, Interferon induction by lymphocytic choriomeningitis viruses correlates with maximum virulence. *J Gen Virol* **57**, 275-283 (1981).
12. M. Chen *et al.*, Genomic and biological characterization of aggressive and docile strains of lymphocytic choriomeningitis virus rescued from a plasmid-based reverse-genetics system. *J Gen Virol* **89**, 1421-1433 (2008).
13. W. V. Bonilla *et al.*, Heterologous arenavirus vector prime-boost overrules self-tolerance for efficient tumor-specific CD8 T cell attack. *Cell Rep Med* **2**, 100209 (2021).
14. M. Battegay *et al.*, Quantification of lymphocytic choriomeningitis virus with an immunological focus assay in 24- or 96-well plates. *J Virol Methods* **33**, 191-198 (1991).
15. P. Seiler *et al.*, Induction of protective cytotoxic T cell responses in the presence of high titers of virus-neutralizing antibodies: implications for passive and active immunization. *J Exp Med* **187**, 649-654 (1998).
16. M. Sahin *et al.*, Antibody bivalency improves antiviral efficacy by inhibiting virion release independently of Fc gamma receptors. *Cell Rep* **38**, 110303 (2022).
17. Y. I. Ertuna *et al.*, Vectored antibody gene delivery restores host B and T cell control of persistent viral infection. *Cell Rep* **37**, 110061 (2021).

18. A. L. Kastner *et al.*, Durable lymphocyte subset elimination upon a single dose of AAV-delivered depletion antibody dissects immune control of chronic viral infection. *Immunity* **58**, 481-498 e410 (2025).
19. P. Dosenovic *et al.*, Anti-HIV-1 B cell responses are dependent on B cell precursor frequency and antigen-binding affinity. *Proc Natl Acad Sci U S A* **115**, 4743-4748 (2018).
20. R. K. Abbott *et al.*, Precursor Frequency and Affinity Determine B Cell Competitive Fitness in Germinal Centers, Tested with Germline-Targeting HIV Vaccine Immunogens. *Immunity* **48**, 133-146 e136 (2018).
21. J. J. Taylor, K. A. Pape, H. R. Steach, M. K. Jenkins, Humoral immunity. Apoptosis and antigen affinity limit effector cell differentiation of a single naive B cell. *Science* **347**, 784-787 (2015).
22. N. Williams, N. Kraft, K. Shortman, The separation of different cell classes from lymphoid organs. VI. The effect of osmolarity of gradient media on the density distribution of cells. *Immunology* **22**, 885-899 (1972).
23. B. Eschli *et al.*, Early antibodies specific for the neutralizing epitope on the receptor binding subunit of the lymphocytic choriomeningitis virus glycoprotein fail to neutralize the virus. *J Virol* **81**, 11650-11657 (2007).
24. N. H. Kalhoro, J. Veits, S. Rautenschlein, G. Zimmer, A recombinant vesicular stomatitis virus replicon vaccine protects chickens from highly pathogenic avian influenza virus (H7N1). *Vaccine* **27**, 1174-1183 (2009).
25. A. Dobin *et al.*, STAR: ultrafast universal RNA-seq aligner. *Bioinformatics* **29**, 15-21 (2013).
26. Y. Liao, G. K. Smyth, W. Shi, featureCounts: an efficient general purpose program for assigning sequence reads to genomic features. *Bioinformatics* **30**, 923-930 (2014).
27. W. Huber *et al.*, Orchestrating high-throughput genomic analysis with Bioconductor. *Nat Methods* **12**, 115-121 (2015).
28. C. W. Law, Y. Chen, W. Shi, G. K. Smyth, voom: Precision weights unlock linear model analysis tools for RNA-seq read counts. *Genome Biol* **15**, R29 (2014).
29. R. Liu *et al.*, Why weight? Modelling sample and observational level variability improves power in RNA-seq analyses. *Nucleic Acids Res* **43**, e97 (2015).
30. K. V. Ballman, D. E. Grill, A. L. Oberg, T. M. Therneau, Faster cyclic loess: normalizing RNA arrays via linear models. *Bioinformatics* **20**, 2778-2786 (2004).
31. D. Wu, G. K. Smyth, Camera: a competitive gene set test accounting for inter-gene correlation. *Nucleic Acids Res* **40**, e133 (2012).
32. A. S. Castanza *et al.*, Extending support for mouse data in the Molecular Signatures Database (MSigDB). *Nat Methods* **20**, 1619-1620 (2023).

Figure S1

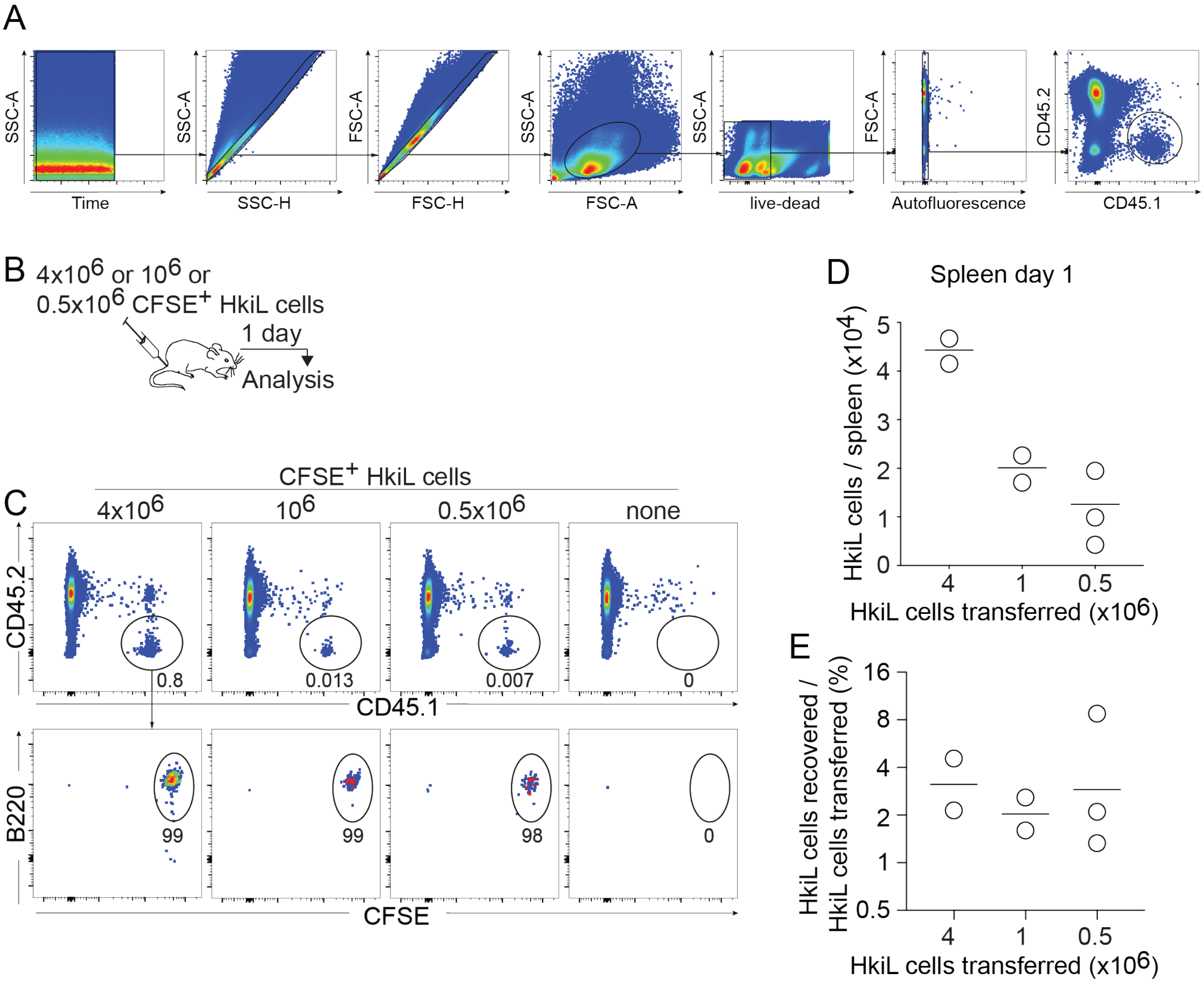

Figure S1: FACS gating strategy for the analysis of adoptively transferred HkiL cells and their splenic take.

A: Gating strategy for the analysis of adoptively transferred HkiL cells.

B-E: We transferred either $4x10^6$, 10^6 or or $5x10^5$ CFSE-labelled HkiL cells on d0 and counted their numbers in spleen 24 hours later (B). Representative FACS plots (C; 2 mice receiving $4x10^6$, 2 mice receiving 10^6 cells and 3 mice receiving $5x10^5$ HkiL cells) pre-gated on live lymphocytes (top) as shown in (A), with the gated HkiL cells analyzed for expression of B220 and CFSE (bottom), documenting homogenously high CFSE levels and uniform B220 expression, as expected for naïve B cells. Total live HkiL cells (D) and the splenic take (E) calculated as (number of HkiL cells recovered from spleen / number of HkiL cells transferred).

Numbers in FACS plots indicate the percentage of gated cells as the group mean (C). Symbols in D and E show individual mice with the mean indicated.

Figure S2

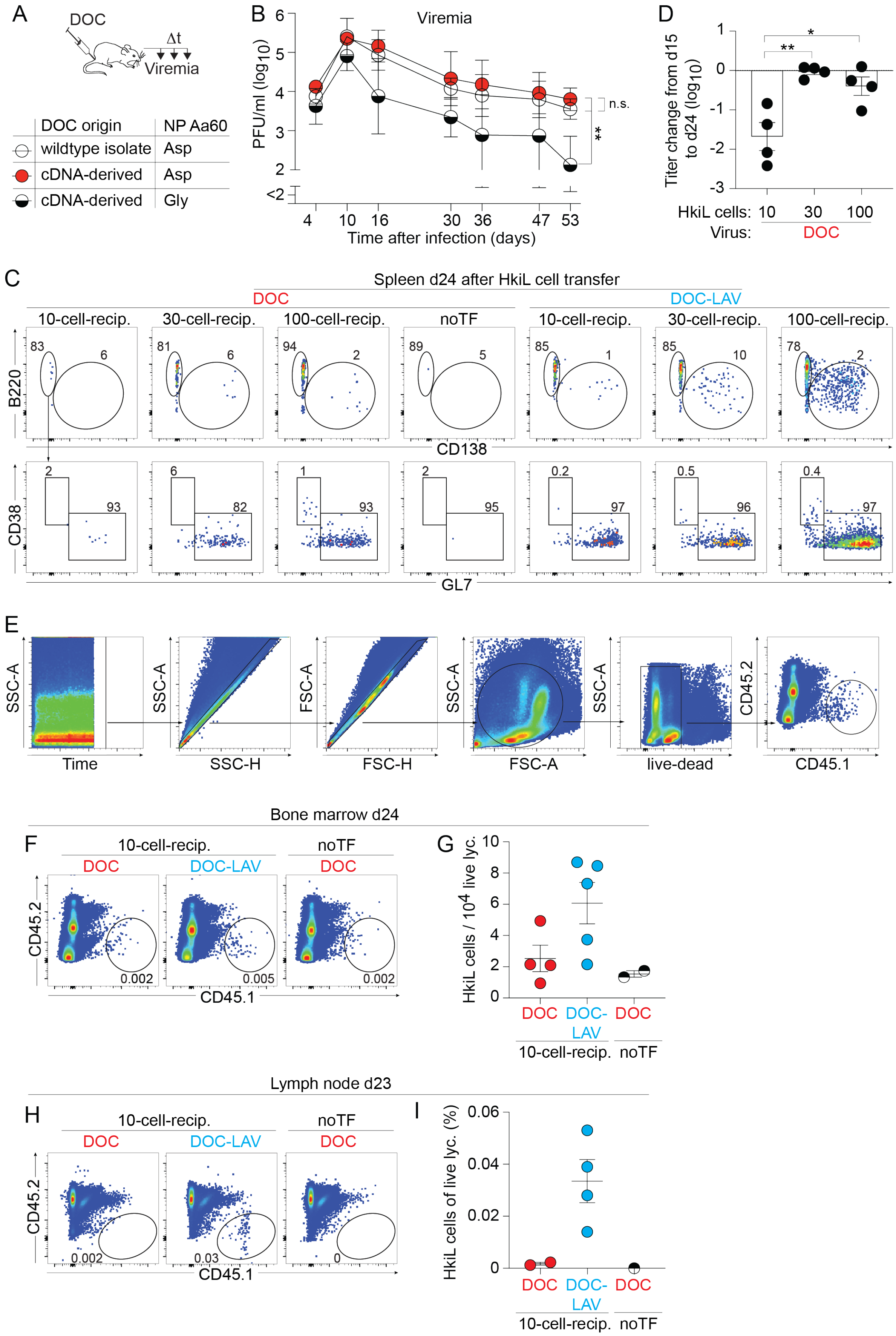
A
DOC
Δt
Viremia
DOC origin | NP Aa60
wildtype isolate | Asp
cDNA-derived | Asp
cDNA-derived | Gly
B
Viremia
PFU/ml (log10)
Time after infection (days)
n.s.
**
D
Titer change from d15 to d24 (log10)
HkiL cells: 10 30 100
Virus: DOC
C
Spleen d24 after HkiL cell transfer
DOC
DOC-LAV
10-cell-recip.
30-cell-recip.
100-cell-recip.
noTF
B220
CD138
CD38
GL7
E
SSC-A
Time
SSC-H
FSC-A
FSC-H
FSC-A
live-dead
CD45.2
CD45.1
Bone marrow d24
F
G
HkiL cells / 10^4 live lyc.
Lymph node d23
H
I
HkiL cells of live lyc. (%)

Figure S2: DOC reverse genetic system, differentiation phenotype of HkiL cells undergoing attrition and manifestation of attrition in lymph nodes and bone marrow.

A-B: Based on cDNAs previously published (12) and following validated techniques (10, 13) we established a reverse genetic system for DOC. We noticed, however, that when administered to mice, the cDNA-derived virus did not establish viremia at levels and of a durability comparable to our wildtype isolate DOC. We therefore revisited the viruses' sequence and identified a coding difference (G60N) in the viral nucleoprotein (NP). Hence, we reverted Aa60 in the NP ORF of our cDNA rescue system to asparagine and generated the corresponding cDNA-derived DOC virus. We then infected mice with either one of three versions of DOC (A,B): wildtype isolate DOC, cDNA-derived DOC with NP Aa60 Asp or cDNA-derived DOC with NP Aa60 Gly. We collected blood over time and determined viremia (B). Reverting NP Aa60 in the cDNA-derived DOC from Gly to Asp restored its ability to establish viremia at levels and with a durability indistinguishable from the wildtype DOC isolate. The DOC and DOC-LAV viruses used in the remainder study have Asp at Aa60 of NP.

C-G: In the experiment to Fig. 2 we infected mice with DOC or DOC-LAV on d-6. On d0 we engrafted either 10, 30 or 100 HkiL cells or none (noTF) and analyzed their progeny in spleen on d24 (C). HkiL cells gated as shown in Fig. 2B were analyzed for B220 and CD138 expression (top), and the $B220^{+}CD138^{-}$ B cell subset was further analyzed for $GL7^{+}CD38^{-}$ GC B cells and $GL7^{-}CD38^{+}$ memory B cells (bottom). The change in KL25 titers from d15 to d24, calculated as (d24 titer minus d15 titer), is shown for DOC-infected mice (D). Each symbol represents an individual mouse, bars show the mean±SEM. From the 10-cell-recipient groups we further enumerated HkiL cell progeny in the bone marrow (F,G), based on the gating strategy shown in (E). Representative FACS plots are shown (F) and the relative abundance in bone marrow (G) was analyzed.

H,I: In an experiment conducted analogously to the one in (C-G) we analyzed HkiL progeny in inguinal lymph nodes on d23 after transferring 10 HkiL cells. Representative FACS plots (H; n=2 in DOC, n=4 in DOC-LAV-infected groups, n=1 in noTf group), pre-gated on live lymphocytes (Fig. S1A) and the relative abundance of HkiL cells (I) are shown. The lack of a detectable HkiL progeny population in lymph nodes of DOC-infected mice was independently confirmed in four mice from one of the experiments reported in Fig. 3J-M. Numbers in FACS plots indicate the percentage of gated cells as the group mean. Symbols in (B) show the mean±SEM of 3-5 mice per group. Symbols and bars in G,I show individual mice with the mean±SEM indicated.

Viremia on d53 in (B) was compared by performing one-way ANOVA with Tukey's post-test. Data in A-I show one representative of two similar experiments.

**: $p<0.01$; n.s.: not statistically significant.

Figure S3

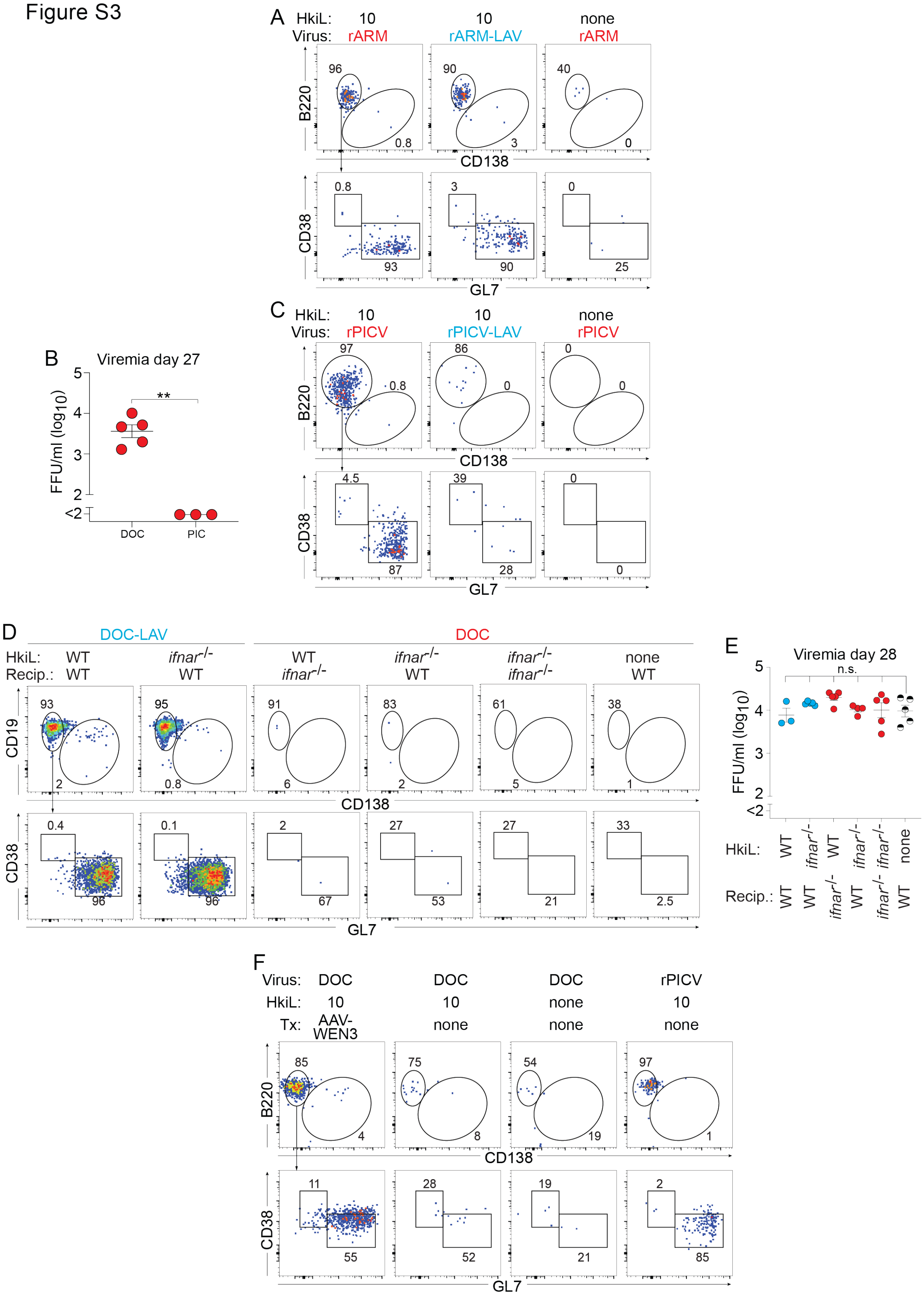

A
HkiL: 10 10 none
Virus: rARM rARM-LAV rARM
96 0.8 90 3 40 0
B220
CD138
0.8 93 3 90 0 25
CD38
GL7
B
Viremia day 27
FFU/ml (log10)
**
DOC PIC
C
HkiL: 10 10 none
Virus: rPICV rPICV-LAV rPICV
97 0.8 86 0 0 0
B220
CD138
4.5 87 39 28 0 0
CD38
GL7
D
DOC-LAV
DOC
HkiL: WT ifnar-/- WT ifnar-/- ifnar-/- none
Recip.: WT WT ifnar-/- WT ifnar-/- WT
93 2 95 0.8 91 6 83 2 61 5 38 1
CD19
CD138
0.4 96 0.1 96 2 67 27 53 27 21 33 2.5
CD38
GL7
E
Viremia day 28
n.s.
FFU/ml (log10)
HkiL: WT ifnar-/- WT ifnar-/- ifnar-/- none
Recip.: WT WT ifnar-/- WT ifnar-/- WT
F
Virus: DOC DOC DOC rPICV
HkiL: 10 10 none 10
Tx: AAV-WEN3 none none none
85 4 75 8 54 19 97 1
B220
CD138
11 55 28 52 19 21 2 85
CD38
GL7

Figure S3: Differentiation of HkiL cells responding to rARM and rPICV, failure of rPICV to establish viremia, and the impact of IFN-I signaling as well as of AAV-WEN3 therapy on HkiL cell differentiation.

A: In the experiment to Fig. 3A-C we infected mice with rARM or rARM-LAV on d-6 and on d0 engrafted 10 HkiL cells or none (noTf). HkiL progeny in spleen were analyzed on d20. Representative FACS plots (n=4 mice except n=2 mice in noTf group) of HkiL cells as gated in Fig. 3B, analyzed for B220 and CD138 expression (top). The $B220^{+}CD138^{-}$ B cell subset was further analyzed for $GL7^{+}CD38^{-}$ GC B cells and $GL7^{-}CD38^{+}$ memory B cells (bottom).

B: We infected mice with DOC or rPICV, engrafted 10 cells at d0 and analyzed viremia on d27. Symbols show individual mice with the mean±SEM indicated.

C: In the experiment to Fig. 3D-F we infected mice with rPICV or rPICV-LAV on d-6 and on d0 engrafted 10 HkiL cells or none (noTF). HkiL progeny in spleen were analyzed on d27. Representative FACS plots (C; n=4 mice for rPIC, n=3 mice for rPICV-LAV group, n=1 for noTF) of HkiL cells as gated in Fig. 3E, analyzed for B220 and CD138 expression (top). The $B220^{+}CD138^{-}$ B cell subset was further analyzed for $GL7^{+}CD38^{-}$ GC B cells and $GL7^{-}CD38^{+}$ memory B cells (bottom).

D,E: In the experiment to Fig. 3G-I we infected *ifnar*$^{-/-}$ and WT control recipients with DOC or DOC-LAV on d-6. On d0 we engrafted them with either 10 HkiL cells or 10 HkiL-*ifnar*$^{-/-}$ cells or left them without cell transfer (”none”, noTF) in the combinations indicated. HkiL and HkiL-*ifnar*$^{-/-}$ progeny ($CD45.1^{+}$) were analyzed on d28. Representative FACS plots (n=5 except HkiL into DOC-LAV-infected WT mice (n=3) and HkiL-*ifnar*$^{-/-}$ into DOC-infected wt mice (n=4)) of the cells gated in Fig. 3H are shown in (D), analyzed for CD19 and CD138 expression (top). The $CD19^{+}CD138^{-}$ B cell subset was further analyzed for $GL7^{+}CD38^{-}$ GC B cells and $GL7^{-}CD38^{+}$ memory B cells (bottom). Viremia was determined on d28 (E).

F: In the experiment to Fig. 3J-M we infected mice with DOC or rPICV on d-6, and on d0 engrafted 10 HkiL cells or none (noTF) as indicated. On d5 we administered AAV-WEN3 to one DOC-infected group. HkiL progeny in spleen were analyzed on d24. Representative FACS plots (n=9 AAV-WEN3-treated HkiL recipients, n=8 recipients without AAV-WEN3, n=9 rPICV-immunized HkiL recipients, n=5 noTF controls) of HkiL cells as gated in Fig. 3K are shown, analyzed for B220 and CD138 expression (top). The $B220^{+}CD138^{-}$ B cell subset was further analyzed for $GL7^{+}CD38^{-}$ GC B cells and $GL7^{-}CD38^{+}$ memory B cells (bottom).

We performed unpaired Student’s t-test for statistical analysis of the values in (B), one-way ANOVA with Dunnett’s post-test was used in (E) to compare viremia of the various HkiL recipients against the noTf group. Data in (A-E) show one out of two similar experiments, (F) reports results from two combined experiments. **: $p<0.01$; n.s.: not statistically significant.

Figure S4

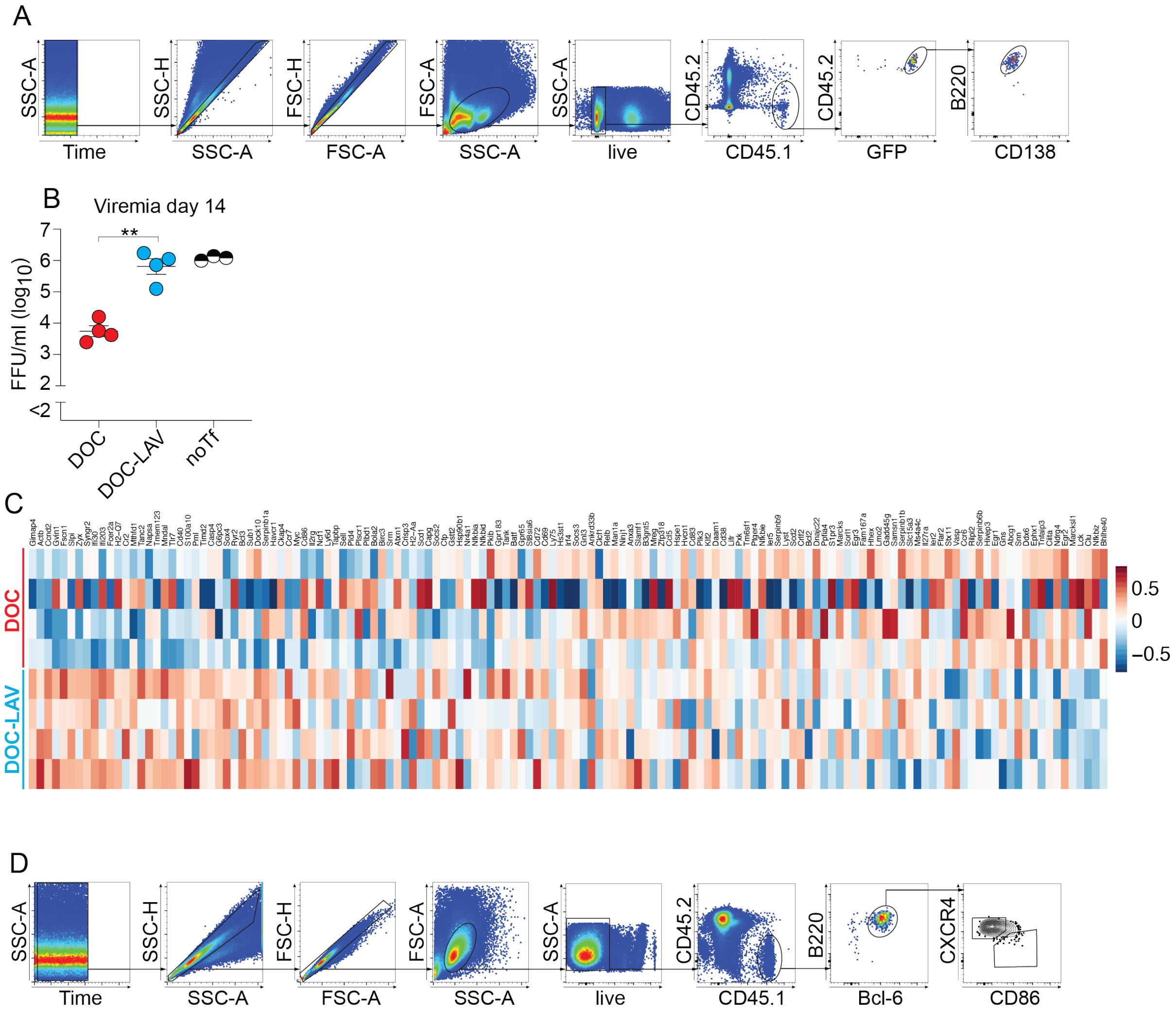

Figure S4: FACS gating strategy for the analysis of adoptively transferred HkiL cells, viremia in DOC- and DOC-LAV-infected HkiL cell recipients, and these cells' expression of GC light zone signature genes.

A: Gating strategy for the sorting of HkiL-GFP cells in the experiment reported on in Figs. 4F-H and 5A-D.

B: Viral titers in the blood of the animals from which HkiL cells were sorted for the analyses reported in Figs. 4F-H and 5A-D. An additional group of DOC-infected mice without HkiL transfer (noTf; not reported on in Figs. 4 or 5) was included for reference. Symbols show individual mice with the mean±SEM indicated. Unpaired Student's t test was performed for statistical analysis. **: p<0.01

C. Heatmaps as shown in Fig. 4G with individual gene names indicated.

D: Gating strategy for the analysis of Bcl6$^+$B220$^+$ HkiL GC B cells in the experiment to Fig. 4I-L.

Figure S5

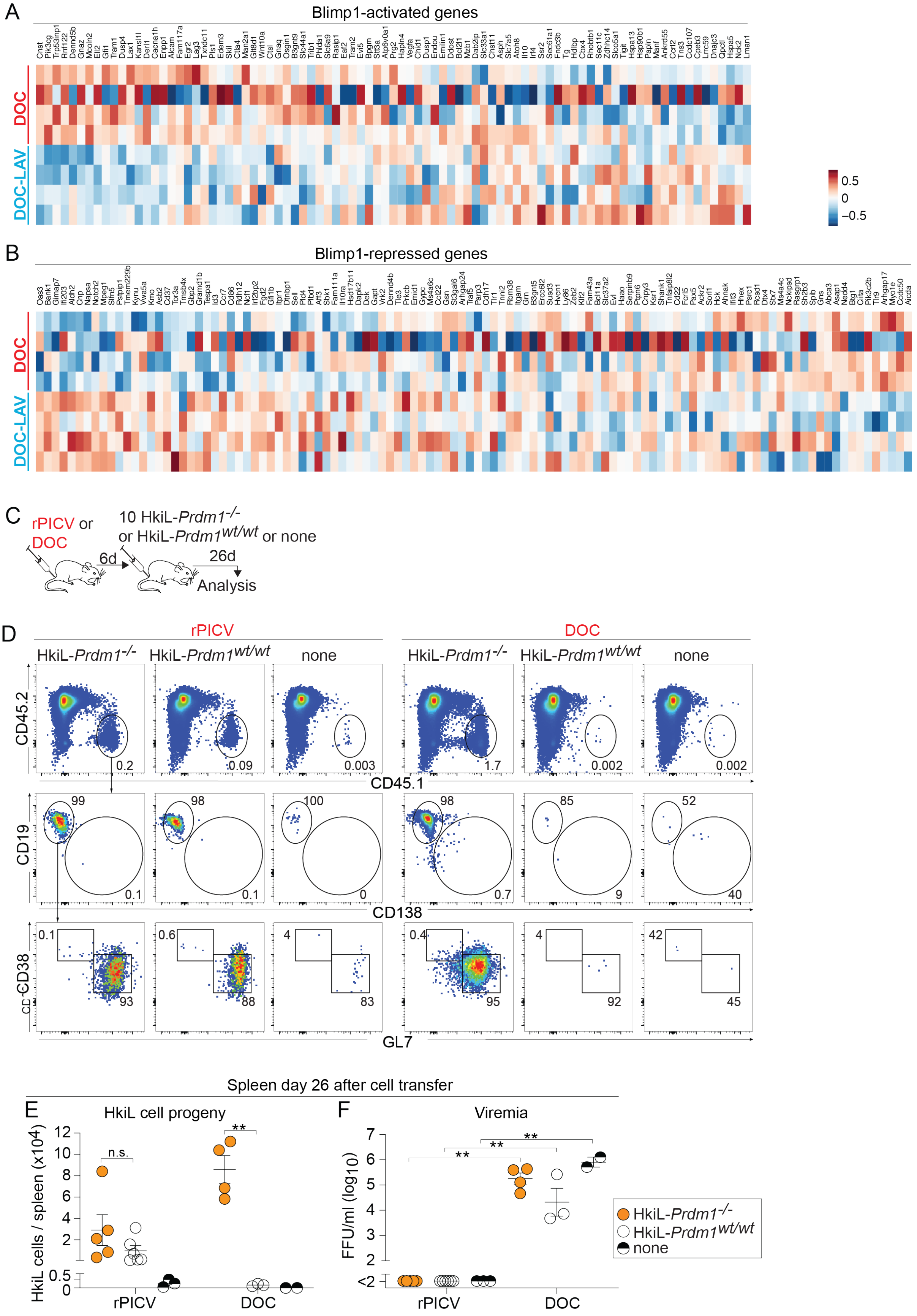

A
Blimp1-activated genes
DOC
DOC-LAV
0.5
0
−0.5
B
Blimp1-repressed genes
DOC
DOC-LAV
C
rPICV or DOC
10 HkiL-Prdm1-/- or HkiL-Prdm1wt/wt or none
6d
26d
Analysis
D
rPICV
DOC
HkiL-Prdm1-/-
HkiL-Prdm1wt/wt
none
CD45.2
CD45.1
CD19
CD138
CD38
GL7
Spleen day 26 after cell transfer
E
HkiL cell progeny
HkiL cells / spleen (x10^4)
n.s.
**
rPICV
DOC
F
Viremia
FFU/ml (log10)
**
rPICV
DOC
HkiL-Prdm1-/-
HkiL-Prdm1wt/wt
none

Figure S5: Differential expression of Blimp-1-regulated genes in HkiL cells responding to DOC and DOC-LAV, and comparable response of *Prdm1*-deficient and -sufficient HkiL cells to rPICV immunization

A,B: Heatmaps as shown in Fig. 5A,C with individual gene names indicated.

C-F: We infected recipient mice with DOC or rPICV on d-6, and on d0 we engrafted either 10 HkiL-*Prdm1*$^{-/-}$ or 10 HkiL-*Prdm1*$^{wt/wt}$ cells or none (noTF). These cells' progeny in spleen were analyzed on d26 (C). Representative FACS plots (D; n=5 rPICV-immunized HkiL-*Prdm1*$^{-/-}$ cell recipients, n=6 rPICV-immunized HkiL-*Prdm1*$^{wt/wt}$ cell recipients, n=3 rPICV-infected noTf mice, n=4 DOC-infected HkiL-*Prdm1*$^{-/-}$ cell recipients, n=3 DOC-infected HkiL-*Prdm1*$^{wt/wt}$ cell recipients, n=2 DOC-infected noTf mice), pre-gated on live lymphocytes (Fig. S1A). HkiL progeny (CD45.1^{+}, top) were analyzed for expression of CD19 and CD138 (center), and their CD19^{+}CD138$^{—}$ subset was analyzed for GL7 and CD38 expression (bottom). HkiL cell progeny numbers (E) and viremia (F) were determined on d26, too.

Numbers in FACS plots indicate the percentage of gated cells as the group's mean. Pair-wise comparisons in (E,F) were performed by unpaired Student's t tests with Bonferroni correction. Results in (D-F) show one representative out of two similar experiments. **: p<0.01; n.s.: not statistically significant.

Table SI

Experiment[1]	HkiL cells engrafted (#)	Mice tested[4]	HkiL cell attrition	Mice (#)	# mice with viral GP Aa119 mutation[2] WT (unmut.)	N119S	N119D	N119K	N119Y	undetermined[3]
Fig. 1K	10	6	No	4	0	3	1	0	0	0
			Yes	2	2	0	0	0	0	0
	100	7	No	7	0	7	0	0	0	0
			Yes	0	n.a.	n.a.	n.a.	n.a.	n.a.	n.a.
	none	1	n.a.	1	1	0	0	0	0	0
Fig. 2H	10	4	No	0	n.a.	n.a.	n.a.	n.a.	n.a.	n.a.
			Yes	4	4	0	0	0	0	0
	30	4	No	4	0	3	0	0	0	1
			Yes	0	n.a.	n.a.	n.a.	n.a.	n.a.	n.a.
	100	4	No	4	0	2	0	1	1	0
			Yes	0	n.a.	n.a.	n.a.	n.a.	n.a.	n.a.
	none	2	n.a.	2	2	0	0	0	0	0

[1] The indicated figure number and legend describe the experiment from which extended data are reported herein

[2] The amino acid at position 119 of the viral GP, which dominates in the blood of individual DOC-infected mice

[3] For one mouse sequencing of the viral GP failed technically

[4] Results related to Fig. 1K summarize two independant experiments, those related to Fig. 2H show one representative of two similar experiments
